# Relationship between ion currents and membrane capacitance in canine ventricular myocytes

**DOI:** 10.1038/s41598-024-61736-6

**Published:** 2024-05-16

**Authors:** Balázs Horváth, Zsigmond Kovács, Csaba Dienes, Zalán Barta, József Óvári, Norbert Szentandrássy, János Magyar, Tamás Bányász, Péter P. Nánási

**Affiliations:** 1 Department of Physiology, Faculty of Medicine, University of Debrecen, Debrecen, Hungary; 2 Faculty of Pharmacy, University of Debrecen, Debrecen, Hungary; 3 Department of Basic Medical Sciences, Faculty of Dentistry, University of Debrecen, Debrecen, Hungary; 4 Division of Sport Physiology, Department of Physiology, Faculty of Medicine, University of Debrecen, Debrecen, Hungary; 5 Department of Dental Physiology and Pharmacology, Faculty of Dentistry, University of Debrecen, Debrecen, Hungary

**Keywords:** Cardiac ion currents, Membrane capacitance, Current densities, Current integrals, Dog myocytes, Action potential voltage clamp, Biophysics, Cell biology, Physiology, Cardiology

## Abstract

Current density, the membrane current value divided by membrane capacitance (C_m_), is widely used in cellular electrophysiology. Comparing current densities obtained in different cell populations assume that C_m_ and ion current magnitudes are linearly related, however data is scarce about this in cardiomyocytes. Therefore, we statistically analyzed the distributions, and the relationship between parameters of canine cardiac ion currents and C_m_, and tested if dividing original parameters with C_m_ had any effect. Under conventional voltage clamp conditions, correlations were high for I_K1_, moderate for I_Kr_ and I_Ca,L_, while negligible for I_Ks_. Correlation between I_to1_ peak amplitude and C_m_ was negligible when analyzing all cells together, however, the analysis showed high correlations when cells of subepicardial, subendocardial or midmyocardial origin were analyzed separately. In action potential voltage clamp experiments I_K1,_ I_Kr_ and I_Ca,L_ parameters showed high correlations with C_m_. For I_NCX_, I_Na,late_ and I_Ks_ there were low-to-moderate correlations between C_m_ and these current parameters. Dividing the original current parameters with C_m_ reduced both the coefficient of variation, and the deviation from normal distribution. The level of correlation between ion currents and C_m_ varies depending on the ion current studied. This must be considered when evaluating ion current densities in cardiac cells.

## Introduction

Ion current density is the membrane current value divided by the measured cell membrane capacitance (C_m_). This process is usually referred to as “normalizing” current values to the obtained C_m,_ which serves as the consensual surrogate measure for the cell surface area. In scientific publications about cellular electrophysiology, ion current magnitudes obtained on different cell populations are expected to be reported and compared based on their ion current densities. This convention assumes linear relationships between magnitudes of ion currents, C_m_, and the cell surface area. In simple terms, bigger cells are expected to generate larger currents. It is widely believed that using current densities instead of absolute current amplitudes decreases the variability of experimental results, and therefore might help in demonstrating real biological differences with statistical methods.

Transmembrane currents, C_m_ and cell surface area are linearly related to each other if (1) the cell membrane composition and (2) the ion channel distribution in the cell membrane is homogeneous, and if (3) C_m_ can be measured accurately. This concept seems to be trivial in small spheroid cells such as red and white blood cells^1,2^, or cells with non-articulated cell surface membrane like neuronal axons^3^. These assumptions, however, are not as intuitive in cardiomyocytes, which are large cells having highly articulated and compartmentalized cell surface membrane with intercalated discs and extensive axial- and transversal tubular network. In fact, the actual relations between transmembrane current, C_m_ and cell surface area in cardiac muscle cells are largely unknown.

Recently, Ismaili et al. studied the relationship between C_m_ and L-type calcium current (I_Ca,L_) amplitude in human and rodent atrial and ventricular cardiomyocytes^4^, while Kula and coworkers performed similar studies with inward rectifier potassium current (I_K1_), acetylcholine-sensitive potassium current (I_K(ACh)_), and transient outward potassium current (I_to_) in rat ventricular cells^5,6^. All these studies reported significant deviations from normal data distribution together with sometimes surprisingly low correlations between current amplitudes and C_m_, with r^2^ ranging from 0.17 to 0.28 in^4^, and r values being 0.04 for I_to_, 0.42 for the constitutively active-, and 0.61 for the acetylcholine-induced component of I_K(ACh),_ while 0.84 for I_K1_^6^.

Computational models and experimental studies indicate that large variations between individual cells exist in ion channel activity that may underlie electrophysiological heterogeneity within the human population^7,8^. Similarly, Ballouz et al. showed large variation in mRNA levels for a wide range of cardiac proteins involved in regulating cellular electrophysiological properties^9^, whereas Lachaud et al. have shown significant inter-cell variability in ventricular APD^10^. Despite these substantial variations in electrophysiological characteristics, bioelectricity of the heart can most likely be properly coordinated because of overlapping functions and certain well-defined correlations between ion currents^11,12^. Most recently, similar relationships in mRNA transcript levels^9^ and in cardiac ion channel co-translation^13^ have also been shown.

In this study, we systematically investigated the relationship between C_m_ and the major cardiac ion currents (L-type calcium current—I_Ca,L_; late sodium current—I_Na,late_; sodium-calcium exchange current—I_NCX_; inward rectifier potassium current—I_K1_; rapid delayed rectifier potassium current—I_Kr_; slow delayed rectifier potassium current—I_Ks_) in canine ventricular myocytes under conventional voltage clamp (CVC) as well as action potential voltage clamp (APVC) conditions. Dogs were chosen because the electrophysiological properties of canine ventricular cells are known to be similar to those of human myocytes^14–16^. Besides investigating a broad range of ion currents, obtained under two different voltage clamp conditions, the novelties of the present study are the comprehensive statistical analyses of (1) the distribution of C_m_ and the ion current parameter values; (2) the relationship between C_m_ and the ion current parameters; and (3) the effect of “normalizing” the original membrane current parameters to C_m_.

We have found generally good correlations between C_m_ and current amplitudes or integrals in this preparation, although correlations were occasionally limited by regional heterogeneity of ion channels and non-ideal experimental conditions.

## Results

### Membrane capacitance

The pooled membrane capacitance (C_m_) values of all cells involved in the study (n = 639) significantly deviated from normal distribution (*p* < 0.001; Supplementary Fig. 1). The distribution was right-skewed (skewness = 0.533) and leptokurtic (excess kurtosis = 0.561). The arithmetic mean of C_m_ was 139.87 ± 1.45 pF, and median value was 139 pF. The observed effect size of the deviation from normal distribution was “small” (φ = 0.228), indicating that the magnitude of the difference between the sample distribution and the normal distribution was small. C_m_ values in any current groups did not deviate significantly from normal distribution, except in I_Ks_ measurements (*p* = 0.013; φ = 0.33; Supplementary Fig. 1J) and when all cells were pooled together in I_to1_ measurements (*p* = 0.036; φ = 0.248; Supplementary Fig. 3A) under CVC experiments.

### L-type Ca^2+^ current (I_Ca,L_)

**Figure 1:**
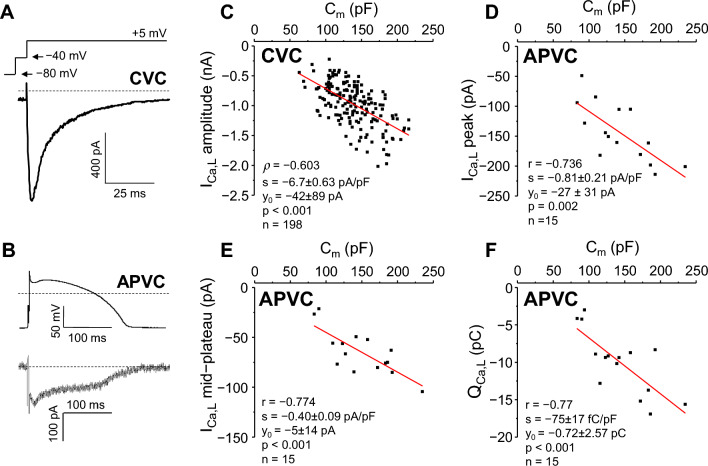
Correlation between C_m_ and I_Ca,L_ in canine ventricular myocytes. Left: representative I_Ca,L_ records obtained under CVC (**A**) and APVC (**B**) conditions, respectively. In both cases command signals are shown above the current records, dashed lines indicate zero voltage and current levels. Right: correlations between C_m_ and I_Ca,L_ peak amplitude measured at + 5 mV under CVC conditions (**C**) and under APVC conditions (**D**), between C_m_ and mid-plateau I_Ca,L_ amplitude (**E**), and between C_m_ and I_Ca,L_ integral (Q_Ca,L_; **F**). Here and in all subsequent figures, red lines were obtained by simple linear regression, where r or ρ indicate the respective correlation coefficient, s is slope of the line (current density or charge density, given as mean ± SEM), y_0_ is the intercept on *y* axis at C_m_ = 0 pF, p is significance of slope, and n is the number of cells analyzed. CVC experiments: 198 cells from 77 animals; APVC experiments: 15 cells from 7 animals.

I_Ca,L_ was studied under CVC conditions in 198 myocytes (Fig. 1A). I_Ca,L_ peak current values (Fig. 1C) showed a significantly non-normal distribution, being left-skewed (towards higher current values; skewness =  − 0.546), with normal kurtosis. The absolute coefficient of variation (CV) of I_Ca,L_ peak was 0.485. The distribution of data still remained significantly non-normal after dividing peak I_Ca,L_ with C_m_, although the CV significantly (*p* = 0.02) reduced to 0.398 after this operation (Supplementary Table 1).

The correlation between the amplitude of I_Ca,L_ and C_m_ was moderate (Spearman’s ρ =  − 0.603). The estimated current density was − 6.7 ± 0.63 pA/pF (Fig. 1C). The close to zero (− 42 ± 89 pA) value of the y intercept supports the actual linear relationship between C_m_ and I_Ca,L_.

Under APVC conditions I_Ca,L_ was dissected as a 1 µM nisoldipine-sensitive current (Fig. 1B) in 15 cells. In contrast to CVC experiments, the distribution of either the I_Ca,L_ current parameters (peak current, mid-plateau current, current integral—Q_Ca,L_) or their values divided with C_m_ did not differ significantly from normal distribution in any case, as determined in APVC experiments (Supplementary Table 2). CVs for these parameters all reduced upon dividing the original values with C_m_, however, changes in CVs did not reach statistical significance.

Pearson’s correlation coefficients were *r* =  − 0.74 and *r* =  − 0.77 for the peak and the mid-plateau values of I_Ca,L_, as shown in Fig. 1D,E, respectively. Similarly, the charge carried by the current, indicated as Q_Ca,L_ (− 75 ± 17 fC/pF) yielded a high correlation with C_m_ (*r* =  − 0.77) (Fig. 1F). These values obtained for I_Ca,L_ density and Q_Ca,L_ are in a good agreement with earlier results obtained in canine ventricular myocytes^16^.

### Late Na^+^ current (I_Na,late_)

**Figure 2:**
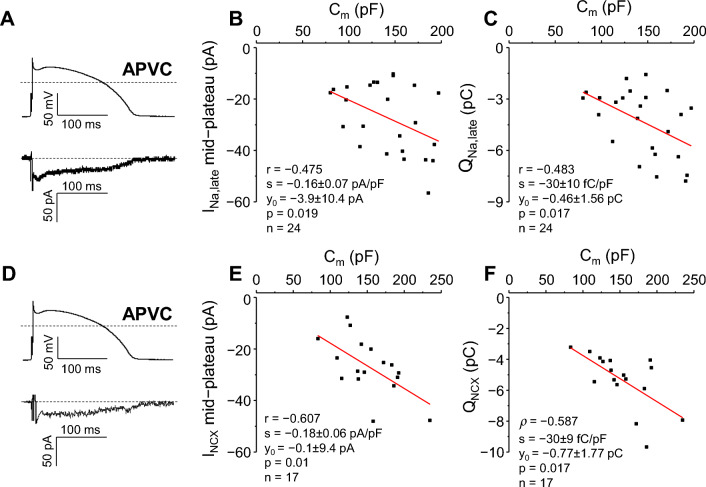
Correlation between C_m_ and I_Na,late_ (**A-C**), and between C_m_ and I_NCX_ (**D-F**). Left: representative I_Na,late_ (**A**) and I_NCX_ (**D**) records obtained under APVC conditions. Command action potentials are shown above the current traces, dashed lines indicate zero voltage and current levels. Right: **B, E**: correlations between C_m_ and mid-plateau currents; **C, F**: correlations between C_m_ and I_Na,late_ and I_NCX_ current integrals (Q_Na,late_; Q_NCX_). I_Na,late_ experiments: 24 cells from 10 animals, I_NCX_ experiments: 17 cells from 8 animals.

I_Na,late_ was recorded exclusively under APVC conditions as a 1 µM GS-458967-sensitive current (Fig. 2A). Since its peak often did not separate from the decaying phase of I_Na,early_, only mid-plateau amplitudes and current integrals (Q_Na,late_) were calculated and analyzed, as shown in Fig. 2B,C and in Supplementary Tables 3 and 6. Both the mid-plateau current values (*p* = 0.029, φ = 0.364) and the total charge carried by the current (*p* = 0.037, φ = 0.392) were significantly non-normally distributed, with medium φ effect sizes, and CVs of 0.493 and 0.447, respectively. After dividing the original values with C_m_, both distributions (mid-plateau I_Na,late_/C_m_ and Q_Na,late_/C_m_) became normal, with their CVs becoming non-significantly smaller (0.415 and 0.373, respectively).

Bivariate distributions of C_m_ and mid-plateau I_Na,late_, or C_m_ and Q_Na,late_ data pairs were not significantly different from the normal bivariate distribution. Pearson’s correlation coefficients were r =  − 0.475 for C_m_ and mid-plateau I_Na,late_, and r =  − 0.483 for C_m_ and Q_Na,late_, respectively, indicating a low linear correlation of mid-plateau I_Na,late_, and Q_Na,late_ with C_m_.

### Na^+^/Ca^2+^ exchanger current (I_NCX_)

I_NCX_ was recorded exclusively under APVC conditions as a 0.5 µM ORM-10962-sensitive current (Fig. 2D). Since its peak often did not separate well from the capacitive transient, we also present only mid-plateau current amplitudes and current integrals (Q_NCX_) here (Fig. 2E,F; Supplementary Tables 3 and 6).

Both original current values and current densities of mid-plateau I_NCX_ were normally distributed, with CVs of 0.403 for original values, and 0.348 for current densities, respectively. Q_NCX_ was significantly non-normally distributed with a large effect size (*p* = 0.024, φ = 0.613), being left-skewed (towards higher current values; skewness =  − 1.274). The distribution became normal after dividing Q_NCX_ with C_m_ (*p* = 0.172), with CVs being 0.311 and 0.227, respectively.

Bivariate distribution of C_m_ and mid-plateau I_NCX_ data pairs was normal, whereas distribution of C_m_ and Q_NCX_ data pairs was significantly different from normal (*p* = 0.049). Correlation coefficients were r =  − 0.607 for C_m_ and mid-plateau I_NCX_, and ρ =  − 0.587 for C_m_ and Q_NCX_, respectively, indicating moderate linear correlation between C_m_ and mid-plateau I_NCX_, and moderate monotonic correlation between C_m_ and Q_NCX_.

### Inward rectifier K^+^ current (I_K1_)

**Figure 3:**
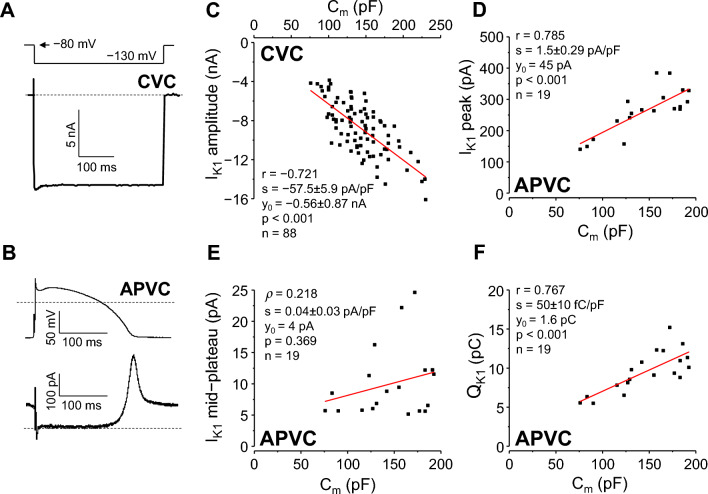
Correlation between C_m_ and I_K1_. Left: representative inward I_K1_ current recorded at − 130 mV under CVC conditions (**A**) and outward I_K1_ record under APVC conditions (**B**). Command signals are shown above the current records, dashed lines indicate zero voltage and current levels. Right: correlations between C_m_ and I_K1_ amplitude, measured at the end of a 400 ms long hyperpolarization to − 130 mV under CVC conditions (**C**), peak amplitude of I_K1_ (**D**), mid-plateau amplitude of I_K1_ (**E**), and I_K1_ integral (Q_K1_; **F**) under APVC conditions. CVC experiments: 88 cells from 35 animals; APVC experiments: 19 cells from 11 animals.

Both original I_K1_ peak (Fig. 3A) values and I_K1_ peak current densities showed normal distribution (with CVs being 0.309 and 0.246, respectively) under CVC conditions. The Forkman’s test showed a significantly (*p* = 0.0495) reduced CV after calculating I_K1_ peak current densities (Supplementary Table 1).

Bivariate distributions of C_m_ and I_K1_ peak amplitude data pairs were normal. These parameters highly correlated with each other (r =  − 0.72, Fig. 3C, Supplementary Table 4). Linear regression analysis yielded a slope of − 57.5 ± 5.9 pA/pF.

Under APVC conditions I_K1_ was defined as a 50 µM BaCl_2_-sensitive current (Fig. 3B) in 19 myocytes. I_K1_ current peaks as well as charges carried by I_K1_ (Q_K1_) were normally distributed in all cases of original values and after dividing them with C_m_. CVs were 0.27 for I_K1_ peak current and 0.162 for I_K1_ peak current density, and 0.276 for Q_K1_ and 0.162 for Q_K1_/C_m_, respectively. Dividing original current parameters with C_m_ significantly reduced data variability in case of I_K1_ (*p* = 0.044 for peak current; *p* = 0.036 for Q_K1_). Both the original mid-plateau I_K1_ values (p < 0.001) and mid-plateau I_K1_ densities (*p* = 0.016) showed significantly non-normal distribution, being right-skewed (towards larger values; skewnesses of 1.569 and 1.003, respectively), with CV values of 0.564 and 0.501, respectively. Mid-plateau I_K1_ densities, however, had only “medium” effect size of the non-normal distribution (φ = 0.442) compared to the “large” effect size (φ = 0.738) in case of the original mid-plateau I_K1_ values (Supplementary Table 3).

Bivariate distributions of C_m_ and I_K1_ peak as well as C_m_ and Q_K1_ data pairs were normal, whereas the bivariate distribution of C_m_ and mid-plateau I_K1_ was significantly different from normal (*p* < 0.001). Correlations between C_m_ and I_K1_ peak (r = 0.785, *p* < 0.001), as well as C_m_ and Q_K1_ (r = 0.767, *p* < 0.001) data pairs were high; but for C_m_ and mid-plateau I_K1_, no significant correlation was detected (see Fig. 3D–F and also Supplementary Table 6). Linear regression analysis between I_K1_ peak current and C_m_ yielded a slope of 1.5 ± 0.29 pA/pF for the regression line, a value close to what has been reported as peak I_K1_ current density in canine ventricular cells^14,16^.

### Rapid delayed rectifier K^+^ current (I_Kr_)

**Figure 4:**
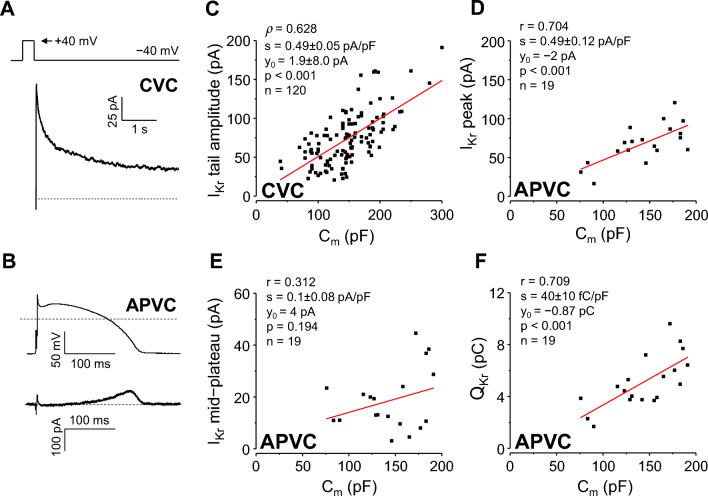
Correlation between C_m_ and I_Kr_. Left: representative I_Kr_ tail current recorded at − 40 mV under CVC conditions (**A**) and an I_Kr_ current trace under APVC conditions (**B**). Command signals are shown above the current records, dashed lines indicate zero voltage and current levels. Right: correlations between C_m_ and I_Kr_ tail current amplitude under CVC conditions (**C**), peak I_Kr_ amplitude (**D**), mid-plateau I_Kr_ amplitude (**E**), and I_Kr_ integral (Q_Kr_; **F)** under APVC conditions. CVC experiments: 120 cells from 54 animals; APVC experiments: 19 cells from 10 animals.

Under CVC conditions both the original I_Kr_ peak current (Fig. 4A) value and I_Kr_ peak current density distributions deviated significantly from normal distribution (*p* < 0.001 and *p* = 0.013, respectively), being right-skewed (towards larger current values; skewness values of 0.904 and 0.548, respectively), with CVs being 0.458 (absolute) and 0.378 (normalized; *p* = 0.075). After “normalizing” to C_m_, the effect size of non-normal distribution changed from medium (φ = 0.379) to small (φ = 0.221). Similarly, bivariate distribution of C_m_ and I_Kr_ peak data pairs differed significantly from the normal distribution. Based on the results obtained in 120 myocytes the Spearman rank-correlation between I_Kr_ tail current amplitude and C_m_ was moderate (ρ = 0.628, *p* < 0.001; Fig. 4C). Linear regression analysis yielded a slope of 0.49 ± 0.05 pA/pF.

Under APVC conditions I_Kr_ peaked during terminal repolarization (Fig. 4B). Contrary to CVC results, when I_Kr_ was investigated with APVC (n = 19), all the measured parameters of the current, as well as their respective bivariate distributions with C_m_ followed normal distribution. The respective CV values for original and C_m_ “normalized” data were 0.359 and 0.266 for I_Kr_ peak; 0.632 and 0.58 for mid-plateau I_Kr_; whereas 0.397 and 0.289 for the charge carried by I_Kr_ (Q_Kr_). Although dividing the original current parameter values with C_m_ reduced CVs, these changes did not reach statistical significance.

High correlations for both I_Kr_ peak amplitude (r = 0.704, *p* < 0.001) and for Q_Kr_ (r = 0.709, *p* < 0.001) were obtained under these conditions (Fig. 4D,F). Similar to I_K1_, the mid-plateau amplitude of I_Kr_ showed no significant correlation with C_m_ (r = 0.312, *p* = 0.194, Fig. 4E). Linear regression analysis showed slope values of I_Kr_ current peak (0.49 ± 0.12 pA/pF) and Q_Kr_ (40 ± 10 fC/pF) similar to the current density and charge density values reported earlier for canine ventricular cells under APVC conditions^16^.

### Slow delayed rectifier K^+^ current (I_Ks_)

**Figure 5:**
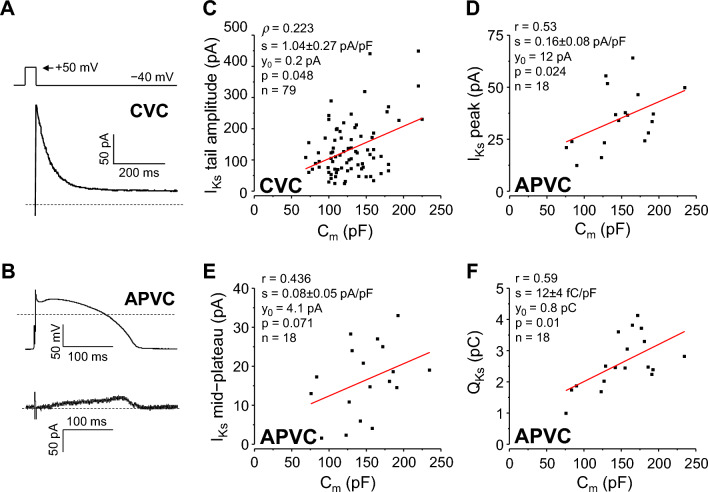
Correlation between C_m_ and I_Ks_. Left: representative I_Ks_ tail current recorded at − 40 mV under CVC conditions (**A**) and I_Ks_ current under APVC conditions (**B**). Command signals are shown above the current records, dashed lines indicate zero voltage and current levels. Right: correlations between C_m_ and I_Ks_ tail current amplitude under CVC conditions (**C**), peak I_Ks_ amplitude (**D**), mid-plateau I_Ks_ amplitude (**E**), and I_Ks_ integral (Q_Ks_; **F)** under APVC conditions. CVC experiments: 79 cells from 27 animals; APVC experiments: 18 cells from 9 animals.

In case of I_Ks_ experiments (Fig. 5A), the membrane capacitance significantly deviated from normal distribution (*p* = 0.013), with a medium effect size (φ = 0.33) when analyzing the results obtained from 79 myocytes under CVC conditions. The distribution was right-skewed (skewness = 0.765).

Both the original and C_m_ “normalized” I_Ks_ peak current value distributions deviated significantly from normal distribution (*p* < 0.001 for both cases), being right-skewed (towards larger current values; skewness values of 1.448 and 0.886, respectively). Moreover, the absolute I_Ks_ peak values were also significantly leptokurtotic (with heavy tails; excess kurtosis = 2.544). CVs were 0.658 and 0.583, respectively. With “normalization” to C_m_, the effect size of the non-normal distribution was reduced from large (φ = 0.592) to medium (φ = 0.359). Similarly, bivariate distribution of C_m_ and I_Ks_ peak data pairs differed significantly from the bivariate normal distribution. Based on the results obtained the Spearman rank-correlation between I_Ks_ tail current amplitude and C_m_ was low (ρ = 0.223, *p* = 0.048; Fig. 5C), with a slope of 1.04 ± 0.27 pA/pF.

Under APVC conditions, I_Ks_ rose slowly during the action potential plateau (Fig. 5B). Contrary to CVC results, when I_Ks_ was investigated with APVC (n = 18), all the measured current parameters, as well as their respective bivariate distributions with C_m_ followed normal distribution. The respective CV values for original and C_m_ “normalized” data were 0.403 and 0.354 for I_Ks_ peak; 0.561 and 0.568 for mid-plateau I_Ks_; whereas 0.321 and 0.245 for the charge carried by I_Ks_ (Q_Ks_). Both C_m_ and I_Ks_ peak (r = 0.53, *p* = 0.024), as well as C_m_ and Q_Ks_ (r = 0.59, *p* = 0.01) correlated moderately, as demonstrated in Fig. 5D,F. No significant correlation was observed between mid-plateau I_Ks_ value and C_m_ (Fig. 5E). Linear regression analysis yielded slopes of 0.16 ± 0.08 pA/pF for I_Ks_ peak, and 12 ± 4 fC/pF for Q_Ks_, respectively.

### Transient outward K^+^ current (I_to1_)

I_to1_ was studied only under CVC conditions using myocytes (n = 108) isolated from subepicardial (EPI), subendocardial (ENDO) and midmyocardial (MID) layers of the left ventricle. During our “regular” cell isolation method there is no physical separation of the transmural layers of the myocardium, therefore we are not able to distinguish between cells originating from the different myocardial layers. Because of the quite thin EPI and ENDO tissue layers, this method dominantly yields MID cells. However, when we cut off delicate layers of tissue from the epicardial and endocardial surface of the myocardium after the enzymatic digestion process (see in Methods and in^17^), myocytes of known origin (*documented* EPI, ENDO, MID cells; n = 30, n = 15, n = 18, respectively) can be obtained.

If all 108 cells were taken into consideration, C_m_ significantly deviated from normal distribution (*p* = 0.036), being right-skewed (skewness = 0.61). However, the effect size of this deviation was small (φ = 0.248). In documented EPI, ENDO, MID as well as in *presumably* MID cells (all cells, except for EPI and ENDO cells), C_m_ followed normal distribution.

**Figure 6:**
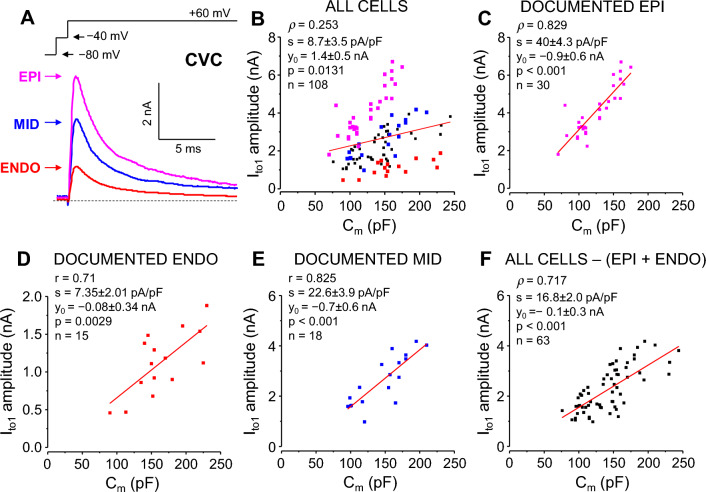
Correlation between C_m_ and I_to1_. (**A**): representative I_to1_ current records obtained at + 60 mV under CVC conditions taken from a subepicardial (EPI; *magenta*), midmyocardial (MID; *blue*), and a subendocardial (ENDO, *red*) myocyte. The command voltage is shown above the current records, the dashed line indicates zero current level. (**B–F**): Correlations between C_m_ and peak I_to1_ currents analyzed in various groups of myocytes. In panel (**B**), all the 108 myocytes from 41 animals were included in the analysis, independent of their origin. In panels (**C**, **D** and **E**) cells with documented EPI (*magenta*), ENDO (*red*), or MID (*blue*) origin were analyzed, respectively, while in panel **F**, the *presumably* MID (non-EPI and non-ENDO cells; *black*) were considered. EPI experiments: 30 cells from 14 animals; ENDO experiments: 15 cells from 10 animals; MID experiments: 18 cells from 13 animals; presumably MID experiments: 63 cells from 36 animals.

As shown in Fig. 6A, marked differences in I_to1_ amplitude were observed among the cells originating from different regions—in line with the known transmural heterogeneity of I_to1_ in canine ventricle^18–20^.

When all cells were analyzed together, both the original and the C_m_ “normalized” I_to1_ peak current distributions deviated significantly from normal distribution (*p* < 0.001 for both cases), with similar “medium” effect sizes (φ = 0.334 and φ = 0.307) and CVs (0.536 and 0.526), respectively. Both distributions were right-skewed (skewness values of 0.837 and 0.752, respectively). Similarly, bivariate distribution of C_m_ and I_to1_ peak data pairs differed significantly from the normal distribution (*p* < 0.001). The Spearman rank-correlation between all I_to1_ peak current amplitudes and C_m_ was low, but statistically significant (ρ = 0.253, *p* = 0.008, Fig. 6B; Supplementary Table 5).

*Documented* ENDO and MID cells shown normal peak current and C_m_ normalized peak current distributions, with CVs being 0.371 *vs* 0.28 (ENDO) and 0.377 *vs* 0.226 (MID) for absolute peak currents, and for normalized peak currents, respectively.

*Documented* EPI cells had normal peak current, but significantly non-normal C_m_ “normalized” peak current (*p* = 0.008) distribution, with a large (φ = 0.775) effect size. CV of I_to1_ peak current density was significantly smaller (0.194) than in case of original peak current magnitudes (0.323). Visualizing the data revealed that the C_m_ normalized peak current distribution was non-normal because of an outlier cell having C_m_ = 80 pF and a peak I_to1_ amplitude of 4399 pA, yielding a current density of 55 pA/pF. If we omitted this outlier from the Shapiro–Wilk test, no significant deviation from the normal distribution could be detected.

Even though there was a clear trend for a reduction in CV after normalizing to C_m_, this effect was only significant in case of EPI cells (*p* = 0.012).

Bivariate distributions of C_m_ and I_to1_ peak data pairs were normal in case of ENDO and MID myocytes, however in case of EPI cells, it significantly deviated from normal (*p* = 0.01), because of the previously mentioned outlier cell. Data in cells of known origin showed strong and statistically significant correlations. Pearson’s correlation coefficients of 0.711 (*p* = 0.003) and 0.825 (*p* < 0.001) were obtained for *documented* ENDO and MID cells, respectively, whereas the Spearman’s correlation coefficient was 0.829 (*p* < 0.001) in EPI myocytes. (Fig. 6C–E).

Myocytes of *presumably* MID origin (all cells except for the *documented* EPI and ENDO cells) showed significantly non-normal peak current (*p* = 0.007, φ = 0.345), but normal peak current density (*p* = 0.081) distributions, with a significantly reduced CV (from 0.386 to 0.266; *p* = 0.009) after “normalization” to C_m_. In these *presumably* MID myocytes, bivariate distribution of C_m_ and I_to1_ peak data pairs significantly deviated from normal (*p* = 0.003). The correlation between C_m_ and I_to1_ peak was also high (ρ = 0.717, *p* < 0.001) in these cells (Fig. 6F). Comparing I_to1_ current densities obtained for the *documented versus* the *presumably* MID cells (22.6 ± 3.9 pA/pF *vs* 16.8 ± 2.0 pA/pF, respectively) confirms the usual assumption that most of the myocytes of undefined origin were likely digested from the midmyocardial layer.

## Discussion

This is the first study to systematically investigate a broad range of ion currents, obtained under two different voltage clamp conditions in canine ventricular myocytes to provide comprehensive statistical analyses of (1) the distribution of C_m_ and the ion current parameter values; (2) the relationship between C_m_ and the ion current parameters; and (3) the effect of “normalizing” the original membrane current parameters to C_m_.

The distribution of C_m_ of all cells involved our study was significantly non-normal, being right-skewed, similar to the findings of Kula et al.^5^. C_m_ values in case of any of the current groups did not deviate significantly from normal distribution, except in I_Ks_ measurements and when all cells were pooled together in I_to1_ measurements under CVC conditions. It is worth noting, however, that all normality tests get more and more sensitive to violations of normality as the sample size gets larger^21^, therefore we have found multiple deviations from normal distribution in our CVC experiments where sample sizes were at least n = 79.

Absolute peak current distributions obtained under CVC conditions were significantly non-normally distributed in case of I_Ca,L_, I_Kr_, I_Ks_, and if all cells were pooled together in I_to1_ experiments (Supplementary Tables 1 and 2). Dividing peak current values with C_m_ (obtaining peak current densities) did not normalize the distributions, all of them remained significantly non-normal. However, effect sizes of the deviation from normal distribution, as well as CV values were smaller in the groups of peak current densities compared to the original peak currents. Reduction in CVs were statistically significant in cases of I_Ca,L_, I_K1_, and I_to1_ (in the EPI and *presumably* MID groups). In their recent study, Kula et al.^5^ also found significantly non-normal, right-skewed I_K1_ and I_K(ACh)_ current magnitude distributions with the Shapiro–Wilk test, and the calculated respective current densities were also significantly non-normally distributed. Ismaili and coworkers^4^ studied distributions of I_Ca,L_ current peaks in various species, under different conditions. Most of their samples did not follow normal distribution and were seemingly right-skewed. When they divided I_Ca,L_ current peaks with C_m_ they had no consistent effect on CV, whereas in our study, the same operation significantly reduced CV in several cases. It is worth noting that in our studies, only 5 cells (less than 1% of all cells investigated) had C_m_ values less than 70 pF, whereas in the previously mentioned two studies^4,5^, a much larger proportion of the left ventricular cells had C_m_ values lower than 70 pF, especially in rat samples. These more frequently occurring low C_m_ values might lead to a greater deviation from normal distribution.

Under APVC conditions, distributions of investigated current parameters did not differ significantly from the normal distribution in most cases, with the exceptions of mid-plateau I_Na,late_, Q_Na,late_, Q_NCX_ and mid-plateau I_K1_ (Supplementary Table 3). Dividing these current parameters with C_m_ “normalized” mid-plateau I_Na,late_, Q_Na,late_ and Q_NCX_ distributions, whereas distribution of the mid-plateau I_K1_ remained significantly non-normal, although the effect size of the deviation from non-normal distribution became much smaller (φ = 0.738 and φ = 0.442, respectively; Supplementary Table 3). In general, CVs were non-significantly reduced after dividing with C_m_, however, in the cases of peak I_K1_ and Q_K1_, these reductions were statistically significant (Supplementary Table 3). In conclusion, we found dividing original data with C_m_ values generally useful, both by reducing the effect of the sometimes originally non-normal sample distributions (even rendering them normal in certain cases), as well as by reducing CVs of the samples.

Relationships between C_m_ and various ion current parameters were studied using *Pearson* and *Spearman* correlations and simple linear regression analysis under both APVC and CVC conditions. In case of many ion currents, including I_Ca,L_, I_K1_ and I_Kr_, the correlation was usually high. Importantly, y intercepts of linear regression were close to zero indicating a true linear relationship between C_m_ and membrane current parameters (I_m_) for these ion currents. This is in line with the general assumption that ion current densities are constant, i.e. current amplitudes are linearly related to cell surface area. On the other hand, moderate correlation was obtained for I_NCX_ and low for I_Na,late_ and I_Ks_.

Marked differences between individual ion currents of the same species regarding the correlation between C_m_ and I_m_ is not exceptional. Good correlation was found in rat myocytes in the case of I_K1_ but not for I_to_^6^—results identical to our observations in canine myocytes. Comparing the present results on canine I_Ca,L_ with those reported by Ismaili et al.^4^ on human ventricular I_Ca,L_ (both obtained under CVC conditions) our correlation coefficient (r =  − 0.603, r^2^ = 0.364) was higher than that reported by Ismaili et al. (r^2^ = 0.28). In that study the low correlation coefficient was attributed partially to inhomogeneous distribution of surface *versus* T-tubular localization of L-type Ca^2+^ channels^22,23^, which channel subpopulations might also be differently regulated^24,25^. This regional inhomogeneity at a cellular level may tremendously increase variability (mainly due to improper voltage control of T-tubular Ca^2+^ channels), however, these effects are likely similar in the cardiomyocytes of a given species.

Another possible reason for the low correlation between C_m_ and I_m_ might be a limited proportionality between the cell size and C_m_^4,6^. Although the exact level of correlation between C_m_ and cell surface is not known, if there was any discrepancy between them, the relationship between C_m_ and I_m_ should have been affected similarly in the case of several ion currents. Furthermore, inaccurate C_m_ measurements^26^ may also contribute to the limited proportionality between I_m_, C_m_, and cell surface area. Again, however, even if there was any inaccuracy in measuring C_m_, it likely had the same systemic effect on all cells. Although we cannot completely rule out that such an inaccuracy could have disproportionally affected certain groups of cells, therefore limiting the correlation between I_m_ and C_m_.

Then, what might limit correlations between I_m_ and C_m_? Theoretically, there are at least three factors which might decrease the linear relationship between C_m_ and ion current amplitudes or integrals. These are: transmural and cell surface inhomogeneity of ion channel expression; small measured current values; and cell-to-cell variability of intracellular ion concentrations, especially [Ca^2+^]_i_.

Our results with I_to1_ represent a good example that regional inhomogeneity of ion channel expression is likely to be a limiting factor in significant correlations between I_m_ and C_m_. I_to1_ is known to be abundantly expressed in the subepicardial layer, its expression is less pronounced in mid-myocardial cells, while the current is small in the subendocardial region of the canine heart^17–20^. Indeed, the correlation between I_to1_ and C_m_ vas negligible (r = 0.21) when all the cells were analyzed independently of their origin. In contrast, the correlation became much better when cells with documented origins were analyzed separately: correlation coefficients of ρ = 0.829, r = 0.711 and r = 0.825 were obtained for subepicardial, subendocardial and midmyocardial cell populations, respectively. However, transmural inhomogeneity has been reported not only for I_to1_, but also for I_Na_, I_NCX_ and I_Ks_. More specifically, the density of I_Na_ is the highest in the mid-myocardial region and significantly lower in the subepicardial and subendocardial layers in dogs^17,27,28^ and marked transmural inhomogeneity was observed in the case of I_NCX_ as well^29,30^. Regarding I_Ks_, the density of the current is higher in the subepicardial than in the midmyocardial region^17,31^. Another type of regional inhomogeneity in the density of I_Ks_ was also observed. Expression of I_Ks_ was found to be more than double in the apical than in the basal region of the canine ventricular wall^32^. In contrast, no transmural inhomogeneity has been observed for I_Ca,L_, I_Kr_ or I_K1_ in canine ventricular myocardium^17^. Similarly, no apico-basal differences regarding the distribution of I_K1_ or I_Kr_ were demonstrated^32^.

Cell surface inhomogeneity of ion channel expression can also limit the correlation between certain currents and C_m_. In differently sized cells, certain membrane compartments may represent different fractions of the total cell surface. For example, based on pure geometrical considerations, in short, wide cells, the relative membrane surface of intercalated discs is likely to be higher than in long, narrow ones. Therefore, the cell membrane of short, wide cells likely contains relatively more ion channels that are preferentially located in the intercalated discs (such as Nav1.5, Kir2.1 and Kir6.2^33^) than long, narrow cells. Finding out these discrepancies, however, are well beyond the scope of the present study.

**Figure 7:**
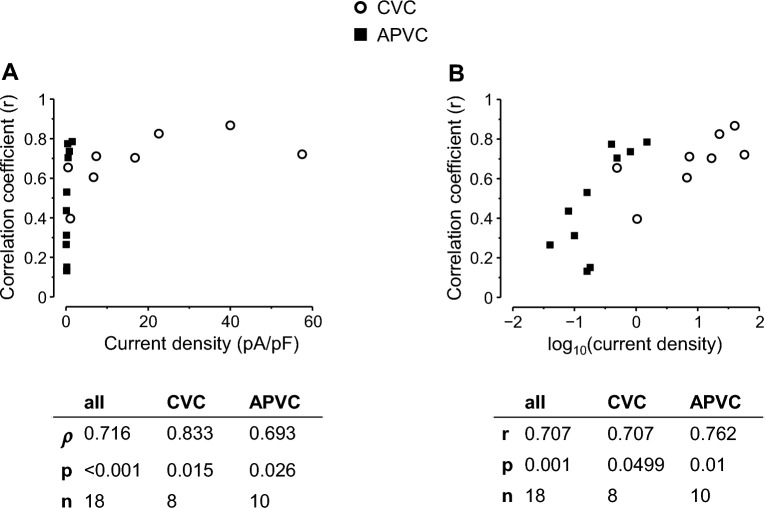
Relationship between Pearson’s correlation coefficients and current densities in case of the studied currents and conditions. Open circles indicate data from conventional voltage clamp (CVC); whereas filled squares show data from action potential voltage clamp (APVC) experiments. (**A**): Pearson’s correlation coefficients between C_m_ and ion current values are plotted against their respective ion current densities yielding significant monotonic (Spearman) correlations between them. (**B**): Pearson’s correlation coefficients between C_m_ and ion current values plotted against the log_10_ values of their respective ion current densities. There are significant linear relationships between these data pairs, suggesting a logarithmic relationship between data pairs of correlation coefficients of C_m_ *versus* ion current values and their respective ion current densities.

An additional source of limited correlations between I_m_ and C_m_ may be the inherent inaccuracy of measurements. This error is expected to be larger when measuring currents with low amplitudes—a problem evident especially under APVC conditions. This issue is illustrated on Fig. 7, where the Pearson’s correlation coefficients obtained between C_m_ and certain ion current parameters are plotted as a function of their respective ion current densities. There were significant monotonic correlations between the Pearson’s correlation coefficients and their respective original current density values (Fig. 7A; ρ = 0.716, *p* < 0.001 for all currents; ρ = 0.833, *p* = 0.015 for CVC; ρ = 0.693, *p* = 0.026 for APVC conditions), whereas after logarithmic transformation of the current density values, significant linear relationships could be seen (Fig. 7B; r = 0.707, *p* = 0.001 for all conditions; r = 0.707, *p* = 0.0499 for CVC; r = 0.762, *p* = 0.01 for APVC conditions). These correlations suggest a logarithmic association between the Pearson’s correlation coefficients and their respective original current density values. This may explain the non-significant correlations between C_m_ and the mid-plateau amplitudes of I_K1_ and I_Kr_, in sharp contrast with the significant and high correlations obtained for their peak amplitudes (r = 0.785 and r = 0.704; p < 0.001 for both) or current integrals (r = 0.767 and r = 0.709; p < 0.001 for both) under APVC conditions.

Inhomogeneity of intracellular Ca^2+^ concentration—a possible consequence of the variable Na^+^ and Ca^2+^ loads occurring during cell isolation—may also affect the correlation in the case of some strongly Ca^2+^-dependent ion currents, such as I_Ca,L_ or I_NCX_, but also I_Na,late_^34^ and I_Ks_^16^. Furthermore, if the turnover of ion channels in the membrane may be fast enough, the time elapsed from cell isolation to the measurement may also influence the amplitude of an ion current^35^. Somewhat related to this, the relatively slow, but continuous reduction of current ion magnitude over time (ion current “run-down”) may also contribute to the limited correlations between I_m_ and C_m_.

Since the experimental conditions were quite uniform in our APVC experiments, their results can be used for evaluation of effects of the above mentioned “burdens” on the correlation coefficients. This is summarized in Table 1, where all APVC experimental arrangements are included. According to these data, both regional inhomogeneity and low current amplitude are likely dominant reasons for limited proportionality between C_m_ and ion current amplitudes. Under APVC conditions it also must be noted that whenever the bivariate distribution of the investigated parameter and C_m_ was significantly (or marginally significantly) non-normal, no significant correlations were detected. These cases included the values of mid-plateau I_Na,late_, mid-plateau I_NCX_, mid-plateau I_Kr_ and mid-plateau I_K1_. These infringements of normal bivariate distributions may arise from the above mentioned “burdens”, such as regional inhomogeneity of ion channel expression, small measured current values and variability of intracellular ion concentrations.

**Table 1:** Burdens that might limit proportionality between ion current amplitudes and cell size under APVC conditions (+ : affected).

Ion current	Parameter	Correlation coefficient	Non-normal distribution?	Regional differences	Low current amplitude
I_Ca,L_	peak	− 0.736	No		
	mid-plateau	− 0.774	No		
	integral	− 0.77	No		
I_Na,late_	mid-plateau	− 0.28	Yes	+	+
	integral	− 0.475	No	+	+
I_NCX_	mid-plateau	− 0.145	Yes	+	+
	integral	− 0.607	No	+	+
I_K1_	peak	0.785	No		
	mid-plateau	0.218	Yes		+
	integral	0.767	No		
I_Kr_	peak	0.704	No		
	mid-plateau	0.312	No (*p* = 0.057)		+
	integral	0.709	No		
I_Ks_	peak	0.53	No	+	+
	mid-plateau	0.436	No	+	+
	integral	0.59	No	+	+

Our experiments performed under APVC conditions allowed the comparison of correlations found between C_m_ and I_m_ with those between C_m_ and current integrals. From this point of view, current integrals produced correlation coefficients at least as good as those obtained for peak current amplitudes. Furthermore, correlation coefficients obtained under APVC conditions were not lower than those estimated in CVC experiments, even though the number of cells analyzed in the APVC experiments was usually much lower than those used under CVC conditions.

In summary, there is a clear tendency that dividing absolute values of ion current parameters with C_m_ reduces both the coefficient of variation, and the deviation from normal distribution, if there was any. In case of most currents, we found significant, moderate-to-high correlations between ion current amplitudes or integrals and C_m_. However, the level of correlation between an ion current and C_m_ may be variable depending on the ion current studied, which must be considered when routinely evaluating ion current densities in cardiac cells.

## Methods

The study is reported in accordance with ARRIVE guidelines (https://arriveguidelines.org). For details regarding experimental animals, cell isolation procedure, and specific description of electrophysiology experiments, please refer to the *Supplementary Material*.

### Animals

Adult mongrel dogs of either sex were anesthetized with intramuscular injections of ketamine hydrochloride (10 mg/kg; Calypsol, Richter Gedeon, Hungary) and xylazine hydrochloride (1 mg/kg; Sedaxylan, Eurovet Animal Health BV, The Netherlands) according to a protocol approved by the local Animal Care Committee (license N^o^: 2/2020/DEMÁB, 9/2015/DEMÁB). All animal procedures conformed to the guidelines from Directive 2010/63/EU of the European Parliament.

### Electrophysiology

Cells were placed in a plexiglass chamber under an inverted microscope, allowing for continuous superfusion with a modified Tyrode solution by gravity flow at a rate of 1–2 ml/min. Under APVC conditions this solution contained (in mM): NaCl 121, KCl 4, CaCl_2_ 1.3, MgCl_2_ 1, HEPES 10, NaHCO_3_ 25, glucose 10, while under CVC conditions it contained: NaCl, 144; KCl, 5; CaCl_2_, 2.5; MgCl_2_, 1.2; HEPES, 5; glucose, 10; both at pH = 7.35 and osmolality of 300 ± 3 mmol/kg. In all experiments, whole cell configuration of the patch clamp technique was applied^36^, where the bath temperature was set to 37 ºC using a temperature controller (Cell MicroControls, Norfolk, VA, USA). Electrical signals were amplified and recorded (Axopatch 200B, MultiClamp 700A or 700B; Molecular Devices, Sunnyvale, CA, USA) under the control of a pClamp 6, 9 or 10 software (Molecular Devices) following analogue–digital conversion (Digidata 1200, 1322A or 1440A, Molecular Devices). Electrodes, having tip resistances of 2–3 MΩ when filled with pipette solution (pH = 7.3 and osmolality of 285 ± 3 mmol/kg), were fabricated from borosilicate glass. The series resistance was usually between 4 and 8 MΩ, and the experiment was discarded if it changed substantially during the measurement. Cell membrane capacitance (C_m_) was determined in each experiment by applying short (15 ms) hyperpolarizations from + 10 to − 10 mV.

### Conventional voltage clamp (APVC)

Experimental protocols applied under CVC conditions and the components of the bathing and pipette solutions are described in the Supplementary Material.

### Action potential voltage clamp (APVC)

APVC experiments were performed according to the methods described previously^37–39^. To avoid the consequences of cell-to-cell variations in AP morphology, the measurements were performed using a “canonic” AP as command signal, instead of the own AP of the cell. This canonic AP was chosen as a representative midmyocardial canine AP characterized by average parameters. Application of uniform command APs made the comparison of the individual current traces easier. In these experiments the pipette solution contained (in mM): K-aspartate 120, KCl 30, MgATP 3, HEPES 10, Na_2_-phosphocreatine 3, EGTA 0.01, cAMP 0.002, KOH 10 at pH = 7.3 with an osmolarity of 285 ± 3 mmol/kg. Ion currents were dissected pharmacologically by using their selective inhibitors. Accordingly, I_Na,late_ was dissected by 1 µM GS-458967, I_NCX_ by 0.5 µM ORM-10962, I_Ca,L_ by 1 µM nisoldipine, I_Kr_ by 1 µM E-4031, I_Ks_ by 0.5 µM HMR-1556, I_K1_ by 50 µM BaCl_2_, and I_to1_ by 100 µM chromanol-293B applied in the presence of 0.5 µM HMR-1556. Each drug-sensitive current was obtained by subtracting the post-drug trace from the pre-drug one. The cells were superfused for 3–5 min with the inhibitor before recording its effect, which record contained 20 consecutive current traces obtained at a cycle length of 0.7 s. These traces were averaged to reduce the noise and the trace-to-trace fluctuations. The dissected currents were evaluated by determining their maximum values (peak currents), their amplitudes measured at the half-duration of the command AP (mid-plateau current values), and finally the total charge carried by the current (current integrals, Q). During the analysis, the initial 10 ms after the AP upstroke was excluded to omit the capacitive transient.

### Statistics

We tested the normality of data distribution with the Shapiro–Wilk test, except for the distribution of C_m_, where the D’Agostino-Pearson omnibus test was applied^40,41^. If there was a significant deviation from the normal distribution, the effect size of non-normality was also calculated as φ values and were categorized as negligible (φ < 0.1) small (0.1 < φ < 0.3), medium (0.3 < φ < 0.5), or large effect (φ > 0.5)^41^. For the description of data variance, we used the absolute value of the coefficient of variation (CV), defined as the standard deviation divided by the absolute value of the arithmetic mean. We used the *F* statistics proposed by Forkman^42^ to test if dividing the original ion current parameter values with C_m_ (calculating ion current densities, or “normalizing”) caused any significant differences between CVs of original and “normalized” data.

For data pairs (eg. C_m_ and current magnitudes or C_m_ and current integrals), normality of the bivariate distribution was tested with the Shapiro–Wilk test for bivariate normality. If the bivariate distribution of data pairs did not differ significantly from the bivariate normal distribution, correlation between the two variables is described with the *Pearson's correlation coefficient* (r) for linear association. In case of data pairs with significantly non-normal bivariate distribution, *Spearman’s correlation coefficient* (ρ*,* “rho”) for monotonic association is reported. The significance of correlation (p) was also calculated. To provide a comprehensive overview of correlation analysis, parameters for both the *Pearson’s* and *Spearman’s* correlations are reported for all investigated ion current parameters in Supplementary Tables 4, 5 and 6. For further information on correlation analysis and the bivariate normal distribution, see the *Supplementary Material*. Significant correlations were categorized according to the calculated coefficient: high (0.9 ≥|*r*|> 0.7 or 0.9 ≥|ρ|> 0.7), moderate (0.7 ≥|*r*|> 0.5 or 0.7 ≥|ρ|> 0.5), low (0.5 ≥|*r*|> 0.3, 0.5 ≥|ρ|> 0.3) or negligible (0.3 >|*r*| or 0.3 >|ρ|)^43^.

Besides correlation analysis, we also performed simple linear regression with C_m_ as the predictor variable, and the different current parameters (peak amplitude, mid-plateau value, current integral) being response variables. Figures show the slope of the fitted line (s) and the y intercept (y_0_), expressed as mean ± SEM values, whereas (n) denotes the number of myocytes studied.

For statistical analyses, we mainly used Jeffreys’s Amazing Statistics Program (JASP; version 0.16.1, Amsterdam, The Netherlands). We also used Origin 2015 (OriginLab Corporation, Northampton, MA, USA) for simple linear regression, and the Statistics Kingdom website^41^ for calculating the D’Agostino-Pearson omnibus test, and the effect size of non-normality (φ). Results were considered statistically significant in case of p < 0.05; in the summary tables, all p < 0.1 values are reported.

### Supplementary Information

Supplementary Information.

## Data Availability

Data underlying this article are available in the Open Science Framework, at 10.17605/OSF.IO/5X428).

## Abbreviations

C_m_: Membrane capacitance
CVC: Conventional voltage clamp
APVC: Action potential voltage clamp
CV: Coefficient of variation
I_K(ACh)_: Acetylcholine-sensitive potassium current
I_Ca__,L_: L-type Ca^2+^ current
I_NCX_: Na^+^/Ca^2+^ exchanger current
I_Na,late_: Late Na^+^ current
I_Kr_: Rapid delayed rectifier K^+^ current
I_Ks_: Slow delayed rectifier K^+^ current
I_K1_: Inward rectifier K^+^ current
I_to1_: Transient outward K^+^ current
Q_x_: The charge carried by ion current “x” (I_x_ integral)
r: Pearson’s correlation coefficient
ρ: Spearman’s correlation coefficient
φ: Effect size
EPI: Subepicardial cells
ENDO: Subendocardial cells
MID: Mid-myocardial cells
