# Supplementary material for: Relationship between ion currents and membrane capacitance in canine ventricular myocytes
Source: Sci Rep. 2024 May 16;14:11241. doi: 10.1038/s41598-024-61736-6 (PMC11099174; doi:10.1038/s41598-024-61736-6)
Supplement: Supplementary file 1 — Supplementary Information. [file 41598_2024_61736_MOESM1_ESM.pdf]

Relationship between ion currents and membrane capacitance in canine ventricular myocytes

Supplementary Material

Methods

In this study, we retrospectively analyzed the relationship between C_m and the major cardiac ion currents based on cellular electrophysiology data obtained by our research group within the last 15 years. Therefore, no further animals were sacrificed for data analyses described in this article.

Animals

Adult mongrel dogs of either sex were anesthetized with intramuscular injections of ketamine hydrochloride (10 mg/kg; Calypsol, Richter Gedeon, Hungary) and xylazine hydrochloride (1 mg/kg; Sedaxylan, Eurovet Animal Health BV, The Netherlands) according to a protocol approved by the local Animal Care Committee (license N°: 2/2020/DEMÁB, 9/2015/DEMÁB). The age range of the animals were 11-26 months, and they weighed 8-17 kg. All animal procedures conformed to the guidelines from Directive 2010/63/EU of the European Parliament on the protection of animals used for scientific purposes. Bilateral palpebral reflex, jaw tone and response to bilateral painful stimuli (toe pinch) were assessed in every two minutes to monitor the depth of anesthesia. The surgical procedure started after the palpebral reflex and the withdrawal response to painful stimuli had been absent on both sides, there had been no immediate respiratory response to painful stimuli and there had been a considerable drop in the jaw tone for at least six minutes (three consecutive assessments).

Isolation of cardiomyocytes

Single canine myocytes were obtained by enzymatic dispersion using the segment perfusion technique, as previously described [1, 2]. A wedge-shaped section of the ventricular wall (supplied typically by the left anterior descending coronary artery) was cannulated, dissected and perfused with a nominally Ca^{2+}-free Joklik solution (Minimum Essential Medium Eagle, Joklik Modification) for 5 min. This was followed by a 30 min perfusion with Joklik solution supplemented with 1 mg/ml collagenase (Type II, Worthington Biochemical Co., Lakewood, NJ, USA; representing final activity of 224 U/ml) and 0.2% bovine serum albumin (Fraction V., Sigma) containing 50 µM Ca^{2+}. Finally, the tissue was minced, the cells were released and

the normal external Ca^{2+} concentration was gradually restored. The cells were stored in Minimum Essential Medium Eagle at 15 °C until use. This procedure yielded dominantly midmyocardial cells for electrophysiological measurements. In some experiments designed for studying I_{to1} under CVC conditions a dermatome (C.R. Bard Inc. Covington, USA) was used to peel off very thin (0.5 mm) subepicardial and subendocardial strips from the left ventricular wall. Similar samples were also dissected from the central layer of midmyocardium (typically 3 mm underneath the epicardium) [3].

Chemicals

All chemicals were purchased from Sigma-Aldrich (St. Louis, MO, USA), except for GS-458967 (MedKoo Biosciences, Morrisville, NC, USA) and HMR-1556 (Tocris Bioscience, Bristol, UK). ORM-10962 was a kind gift from Prof. András Varró (University of Szeged, Szeged, Hungary).

Electrophysiology – conventional voltage clamp

L-type Ca^{2+} current ($I_{Ca,L}$)

$I_{Ca,L}$ was studied under CVC conditions in 198 myocytes. The cells were bathed with Tyrode solution containing 3 mM 4-aminopyridine, 1 μM E-4031 and 1μM HMR-1556 to block I_{to1}, I_{Kr} and I_{Ks}, respectively. In some experiments I_{to1} and I_{Ks} were suppressed by 30 μM chromanol-293B. The pipette solution contained (in mM): KCl, 110; KOH, 40; HEPES, 10; EGTA 10; TEACl, 20; K_2ATP, 3. The membrane potential was held at −80 mV between the pulses applied at a frequency of 0.5 Hz. The test potential lasting for 400 ms at +5 mV was preceded by a short prepulse of 50 ms duration clamped to −40 mV in order to inactivate Na^+ currents The initial 70 ms of a representative $I_{Ca,L}$ record is presented in **Fig. 1.A**.

Inward rectifier K^+ current (I_{K1})

Under CVC conditions I_{K1} was recorded at the end of a hyperpolarizing pulse to −130 mV, lasting for 400 ms and applied from a holding potential of −80 mV at a rate of 0.2 Hz (**Fig. 3.A**). $I_{Ca,L}$ was blocked by 1 μM nisoldipine added to the bathing medium. The pipette solution contained (in mM): K-aspartate, 100; KCl, 45; $MgCl_2$, 1; HEPES, 5; EGTA, 10; K_2ATP, 3. This pipette solution was used when measuring I_{Kr} and I_{Ks} tail currents as well as I_{to1} current amplitudes.

Rapid delayed rectifier K^+ current (I_{Kr})

Under CVC conditions I_{Kr} was activated by a pulse to +40 mV lasting for 200 ms, then the membrane was repolarized to −40 mV to record the I_{Kr} tail current (**Fig. 4.A**). The holding potential was also −40 mV in these experiments. The pulse protocol was repeated at a rate of 0.05 Hz. For the blockade of $I_{Ca,L}$ 1 μM nisoldipine was added to the bathing medium, while I_{Ks} was suppressed by 1 μM HMR-1556.

Slow delayed rectifier K^+ current (I_{Ks})

Under CVC conditions I_{Ks} was activated with a 3 s long voltage pulse to +50 mV, arising from the holding potential of −40 mV. Then the membrane was repolarized to −40 mV, to record I_{Ks} current tails (**Fig. 5.A**). The pulse protocol was repeated at a frequency of 0.1 Hz. In these experiments $I_{Ca,L}$ and I_{Kr} were inhibited with 1 μM nisoldipine and 1 μM E-4031, respectively.

Transient outward K^+ current (I_{to1})

In the investigated 108 myocytes, the current was activated by a 400-ms-long depolarizing step to +60 mV, preceded by a short (10 ms) depolarization to −40 mV to inactivate sodium channels. This pulse protocol, applied at a rate of 0.2 Hz, arose from the holding potential of −80 mV. The bathing medium contained 1 μM nisoldipine to block $I_{Ca,L}$.

Statistical testing – correlation analysis

There are several assumptions that *Pearson's correlation* analysis imposes, one of which is that the continuous variable pairs should follow a bivariate normal distribution. When this assumption is not met, there is disagreement in the statistical literature about whether *Pearson's correlation* is "robust" enough to violations of normality (that is, whether *Pearson's correlation* is still able to provide a valid result even though the data pairs do not follow the bivariate normal distribution). Questioning the "robustness" of *Pearson's correlation* under conditions that violate bivariate normality are based on whether further assumptions and mathematical measures of robustness are met, and whether these additional assumptions are true in everyday practice.

Authors of this paper do not feel themselves mathematically qualified to argue for or against either side. Instead, we report if there is a significant violation of bivariate normal distribution of the parameter pair in question. If the data pairs do not violate the bivariate normal distribution, we report the *Pearson's correlation coefficient* (*r*) in the text. In cases of significantly non-normal bivariate distribution, we took a conservative approach, and report

the *Spearman's correlation coefficient* (*ρ, "rho"*). However, for a comprehensive overview of correlation analysis, both *Pearson's* and *Spearman's* correlations are shown for all C_m and current parameter data pairs in **Supplementary Tables 4., 5.** and **6.**

For further reading on this issue, we refer to statistics handbooks, and the papers of Knief and Forstmeier [4], Edgell and Noon [5] and Havlicek and Peterson [6].

Results

Distributions of the investigated ion current parameters are reported in the form of bar graphs on **Supplemetary Figures 1-6**. Also, summary of descriptive statistics of the ion current parameters are given in **Supplemetary Tables 1-3**.

As mentioned previously, a comprehensive overview of correlation analysis with both *Pearson's* and *Spearman's* correlations are shown for all C_m and current parameter data pairs in **Supplementary Tables 4., 5.** and **6.**

Authors emphasize that the normalized ion current parameters measured during the APVC experiments describe ion currents from a slightly different perspective than current amplitudes measured during CVC experiments. Therefore, for the purpose of this study, parameters (or normalized parameters) of a given ion current obtained under APVC conditions are not directly comparable with peak current values (or peak current densities) in CVC recordings.

Supplementary Table 1. Summary of descriptive statistics of $I_{Ca,L}$, I_{Kr}, I_{Ks} and I_{K1} under conventional voltage clamp conditions. Parameter distributions are visualized on **Supplemental Figure 2** as bar graphs.

Current	Parameter	Significantly non-normal distribution?	CV	Dividing with C_m significantly reduced CV?
$I_{Ca,L}$	C_m	No	0.227	
	peak $I_{Ca,L}$	Yes ($p=0.002$, $\varphi=0.217$)	0.359	
	peak $I_{Ca,L}/C_m$	Yes ($p=0.006$, $\varphi=0.183$)	0.296	Yes ($p=0.015$)
I_{Kr}	C_m	No	0.307	
	peak I_{Kr}	Yes ($p<0.001$, $\varphi=0.379$)	0.458	
	peak I_{Kr}/C_m	Yes ($p=0.013$, $\varphi=0.221$)	0.378	No ($p=0.075$)
I_{Ks}	C_m	Yes ($p=0.013$, $\varphi=0.33$)	0.261	
	peak I_{Ks}	Yes ($p<0.001$, $\varphi=0.592$)	0.658	
	peak I_{Ks}/C_m	Yes ($p<0.001$, $\varphi=0.359$)	0.583	No
I_{K1}	C_m	No	0.238	
	peak I_{K1}	No	0.309	
	peak I_{K1}/C_m	No	0.246	Yes ($p=0.0495$)

Supplementary Table 2. Summary of descriptive statistics of I_{to1} under conventional voltage clamp conditions. Parameter distributions are visualized on **Supplemental Figure 3** as bar graphs.

Region	Parameter	Significantly non-normal distribution?	CV	Dividing with C_m significantly reduced CV?
all cells	C_m	Yes (p=0.036, φ=0.248)	0.268	
	peak I_{to1}	Yes (p<0.001, φ=0.334)	0.536	
	peak I_{to1}/C_m	Yes (p<0.001, φ=0.307)	0.526	No
EPI	C_m	No	0.229	
	peak I_{to1}	No	0.323	
	peak I_{to1}/C_m	Yes (p=0.008, φ=0.775)	0.194	Yes (p=0.012)
ENDO	C_m	No	0.247	
	peak I_{to1}	No	0.371	
	peak I_{to1}/C_m	No	0.28	No
MID	C_m	No	0.247	
	peak I_{to1}	No	0.377	
	peak I_{to1}/C_m	No	0.226	No (p=0.059)
presumably MID	C_m	No	0.265	
	peak I_{to1}	No	0.386	
	peak I_{to1}/C_m	No	0.266	Yes (p=0.009)

Supplementary Table 3. Summary of descriptive statistics the currents under action potential voltage clamp conditions. Parameter distributions are visualized on **Supplemental Figure 4 and 5** as bar graphs.

Current	Parameter	Significantly non-normal distribution?	CV	Dividing with C_m significantly reduced CV?
$I_{Ca,L}$	C_m	No	0.305	
	peak $I_{Ca,L}$	No	0.336	
	peak $I_{Ca,L}/C_m$	No	0.27	No
	mid-plateau $I_{Ca,L}$	No	0.342	
	mid-plateau $I_{Ca,L}/C_m$	No	0.274	No
	$Q_{Ca,L}$	No	0.413	
	$Q_{Ca,L}/C_m$	No	0.284	No
$I_{Na,late}$	C_m	No	0.241	
	mid-plateau $I_{Na,late}$	Yes (p=0.029, φ=0.364)	0.493	
	mid-plateau $I_{Na,late}/C_m$	No	0.415	No
	$Q_{Na,late}$	Yes (p=0.037, φ=0.392)	0.447	
	$Q_{Na,late}/C_m$	No	0.373	No
I_{NCX}	C	No	0.245	
	mid-plateau I_{NCX}	No	0.403	
	mid-plateau I_{NCX}/C_m	No	0.348	No
	Q_{NCX}	Yes (p=0.024, φ=0.613)	0.331	
	Q_{NCX}/C_m	No	0.227	No
I_{Kr}	C	No	0.247	
	peak I_{Kr}	No	0.359	
	peak I_{Kr}/C_m	No	0.266	No
	mid-plateau I_{Kr}	No	0.632	
	mid-plateau I_{Kr}/C_m	No	0.58	No
	Q_{Kr}	No	0.397	
	Q_{Kr}/C_m	No	0.289	No
I_{Ks}	C_m	No	0.276	
	peak I_{Ks}	No	0.403	
	peak I_{Ks}/C_m	No	0.354	No
	mid-plateau I_{Ks}	No	0.561	
	mid-plateau I_{Ks}/C_m	No	0.568	No
	Q_{Ks}	No	0.321	
	Q_{Ks}/C_m	No	0.245	No
I_{K1}	C_m	No	0.255	
	peak I_{K1}	No	0.27	
	peak I_{K1}/C_m	No	0.162	Yes (p=0.044)
	mid-plateau I_{K1}	Yes (p<0.001, φ=0.738)	0.564	
	mid-plateau I_{K1}/C_m	Yes (p=0.016, φ=0.442)	0.501	No
	Q_{K1}	No	0.276	
	Q_{K1}/C_m	No	0.162	Yes (p=0.036)

Supplementary Table 4. Correlations between membrane capacitance and $I_{Ca,L}$, I_{Kr}, I_{Ks} and I_{K1} peak currents under conventional voltage clamp conditions

Parameter	Pearson		Spearman		Significantly non-normal bivariate distribution?
	r	p	ρ	p	
peak $I_{Ca,L}$	−0.605	<0.001	−0.603	<0.001	Yes (p=0.001)
peak I_{Kr}	0.654	<0.001	0.628	<0.001	Yes (p=0.001)
peak I_{Ks}	0.396	<0.001	0.223	0.048	Yes (p<0.001)
peak I_{K1}	−0.721	<0.001	−0.677	<0.001	No

Supplementary Table 5. Correlations between membrane capacitance and I_{to1} peak under conventional voltage clamp conditions

Region	Pearson		Spearman		Significantly non-normal bivariate distribution?
	r	p	ρ	p	
all cells	0.221	0.022	0.253	0.008	Yes (p<0.001)
EPI	0.867	<0.001	0.829	<0.001	Yes (p<0.01)
ENDO	0.711	0.003	0.64	0.01	No
MID	0.825	<0.001	0.807	<0.001	No
presumably MID	0.703	<0.001	0.717	<0.001	Yes (p=0.003)

Supplementary Table 6. Correlations between membrane capacitance and current parameters under action potential voltage clamp conditions

Parameter	Pearson		Spearman		Significantly non-normal bivariate distribution?
	r	p	ρ	p	
peak $I_{Ca,L}$	−0.736	0.002	−0.771	0.001	No
mid-plateau $I_{Ca,L}$	−0.774	<0.001	−0.689	0.006	No
$Q_{Ca,L}$	−0.77	<0.001	−0.664	0.009	No
mid-plateau $I_{Na,late}$	−0.132	0.54	−0.28	0.185	Yes (p=0.003)
$Q_{Na,late}$	−0.475	0.019	−0.424	0.039	No
mid-plateau I_{NCX}	−0.151	0.563	−0.145	0.579	Yes (p=0.046)
Q_{NCX}	−0.607	0.01	−0.559	0.022	No
peak I_{Kr}	0.704	<0.001	0.644	0.004	No
mid-plateau I_{Kr}	0.312	0.194	0.188	0.44	No (p=0.057)
Q_{Kr}	0.709	<0.001	0.702	0.001	No
peak I_{Ks}	0.53	0.024	0.521	0.028	No
mid-plateau I_{Ks}	0.436	0.071	0.488	0.042	No
Q_{Ks}	0.59	0.01	0.55	0.02	No
peak I_{K1}	0.785	<0.001	0.774	<0.001	No
mid-plateau I_{K1}	0.265	0.274	0.218	0.369	Yes (p<0.001)
Q_{K1}	0.767	<0.001	0.781	<0.001	No

Supplementary Figure legends

Suppl. Fig. 1. Distribution of pooled cell membrane capacitance (C) values of all cells involved in the present study (n=639). Capacitance values were grouped into 10-pF bins to show the distribution as a bar graph. The distribution significantly deviated from normal distribution ($p<0.001$), being right-skewed (skewness=0.533) and leptokurtic (excess kurtosis=0.561). The arithmetic mean of C_m was 139.87±1.45 pF, and median value was 139 pF. The observed effect size of the deviation from normal distribution was “small” ($\varphi=0.228$).

Suppl. Fig. 2. Distribution of cell membrane capacitances (C; left panels), peak current amplitudes (I_peak; middle panels), and peak current densities (J_peak; right panels) in conventional voltage clamp experiments of $I_{Ca,L}$ (**A, B, C**), I_{K1} (**D, E, F**), I_{Kr} (**G, H, I**) and I_{Ks} (**J, K, L**).

Suppl. Fig. 3. Distribution of cell membrane capacitances (C; left panels), peak current amplitudes (I_peak; middle panels), and peak current densities (J_peak; right panels) in conventional voltage clamp experiments of I_{to1}. Distributions are shown for all the cells involved in the study (**A, B, C**), for cells of documented subepicardial origin (EPI; **D, E, F**), for cells of documented subendocardial origin (ENDO; **G, H, I**), for cells of documented midmyocardial origin (MID; **J, K, L**) and for the *presumably* MID cells (that is, all cells except for the documented EPI and ENDO cells; **M, N, O**).

Suppl. Fig. 4. Distribution of cell membrane capacitances (**A, F**) and ion current parameters (**B-E, G-J**) for experiments with the late sodium current ($I_{Na,late}$, upper panels) and the sodium-calcium exchange current (I_{NCX}, lower panels), respectively, under action potential voltage clamp conditions. **A, F**: cell membrane capacitances (C) for experiments with $I_{Na,late}$ and $I_{NCX;}$ **B, G**: mid-plateau ion current magnitudes (I_P50%); **C, H**: current integrals (Q); **D, I**: mid-plateau ion current densities (J_P50%); **E, J**: current integrals divided with membrane capacitance (Q/C).

Suppl. Fig. 5. Distribution of cell membrane capacitances (**A, H**) and ion current parameters (**B-G, I-N**) for experiments with the L-type calcium current ($I_{Ca,L}$, upper panels) and the inward rectifying potassium current (I_{K1}, lower panels), respectively, under action potential voltage clamp conditions. **A, H**: cell membrane capacitances (C) for experiments with $I_{Ca,L}$ and $I_{K1;}$ **B, I**: peak ion current amplitudes (I_peak); **C, J**: mid-plateau ion current magnitudes (I_P50%); **D, K**: current integrals (Q); **E, L**: peak ion current densities (J_peak); **F, M**: mid-plateau ion current densities (J_P50%); **G, N**: current integrals divided with membrane capacitance (Q/C).

Suppl. Fig. 6. Distribution of cell membrane capacitances (**A, H**) and ion current parameters (**B-G, I-N**) for experiments with the rapid (I_{Kr}, upper panels) and the slow (I_{Ks}, lower panels) delayed rectifier potassium currents, respectively, under action potential voltage clamp conditions. **A, H**: cell membrane capacitances (C) for experiments with $I_{Ca,L}$ and $I_{K1;}$ **B, I**: peak ion current amplitudes (I_peak); **C, J**: mid-plateau ion current magnitudes (I_P50%); **D, K**: current integrals (Q); **E, L**: peak ion current densities (J_peak); **F, M**: mid-plateau ion current densities (J_P50%); **G, N**: current integrals divided with membrane capacitance (Q/C).

References

[1] B. Hegyi, B. Horvath, K. Vaczi, M. Gonczi, K. Kistamas, F. Ruzsnavszky, R. Veress, L.T. Izu, Y. Chen-Izu, T. Banyasz, J. Magyar, L. Csernoch, P.P. Nanasi, N. Szentandrassy, Ca2+-activated Cl- current is antiarrhythmic by reducing both spatial and temporal heterogeneity of cardiac repolarization, Journal of molecular and cellular cardiology 109 (2017) 27-37.

[2] B. Horvath, K. Vaczi, B. Hegyi, M. Gonczi, B. Dienes, K. Kistamas, T. Banyasz, J. Magyar, I. Baczko, A. Varro, G. Seprenyi, L. Csernoch, P.P. Nanasi, N. Szentandrassy, Sarcolemmal Ca(2+)-entry through L-type Ca(2+) channels controls the profile of Ca(2+)-activated Cl(-) current in canine ventricular myocytes, Journal of molecular and cellular cardiology 97 (2016) 125-39.

[3] G. Szabo, N. Szentandrassy, T. Biro, B.I. Toth, G. Czifra, J. Magyar, T. Banyasz, A. Varro, L. Kovacs, P.P. Nanasi, Asymmetrical distribution of ion channels in canine and human left-ventricular wall: epicardium versus midmyocardium, Pflugers Archiv : European journal of physiology 450(5) (2005) 307-16.

[4] U. Knief, W. Forstmeier, Violating the normality assumption may be the lesser of two evils, Behavior Research Methods 53(6) (2021) 2576-2590.

[5] S. Edgell, S. Noon, Effect of violation of normality on the t test of the correlation coefficient, Psychological Bulletin 95 (1984) 576-583.

[6] L.L. Havlicek, N.L. Peterson, Robustness of the Pearson Correlation against Violations of Assumptions, Perceptual and Motor Skills 43(3_suppl) (1976) 1319-1334.

Suppl. Fig. 1.

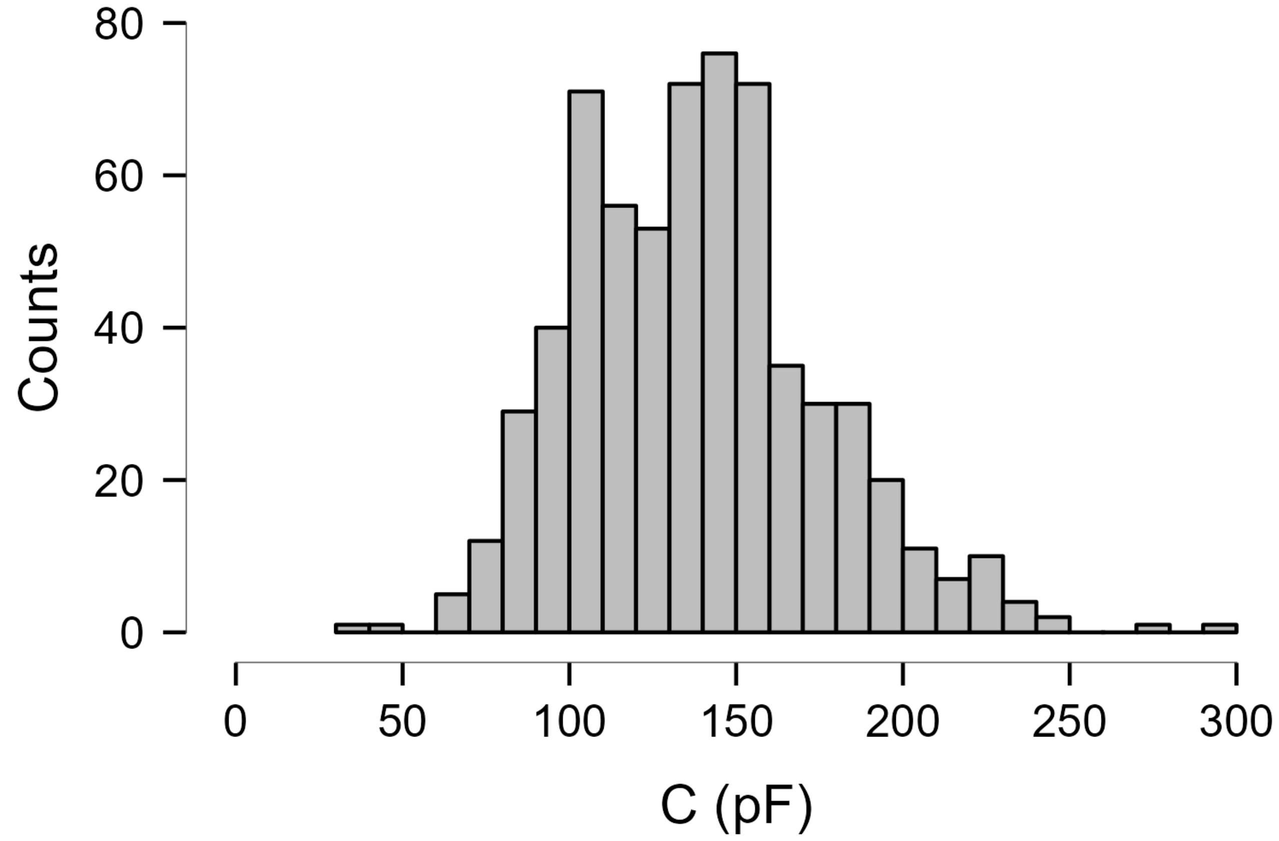

Suppl. Fig. 2.

CVC – $I_{Ca,L}$

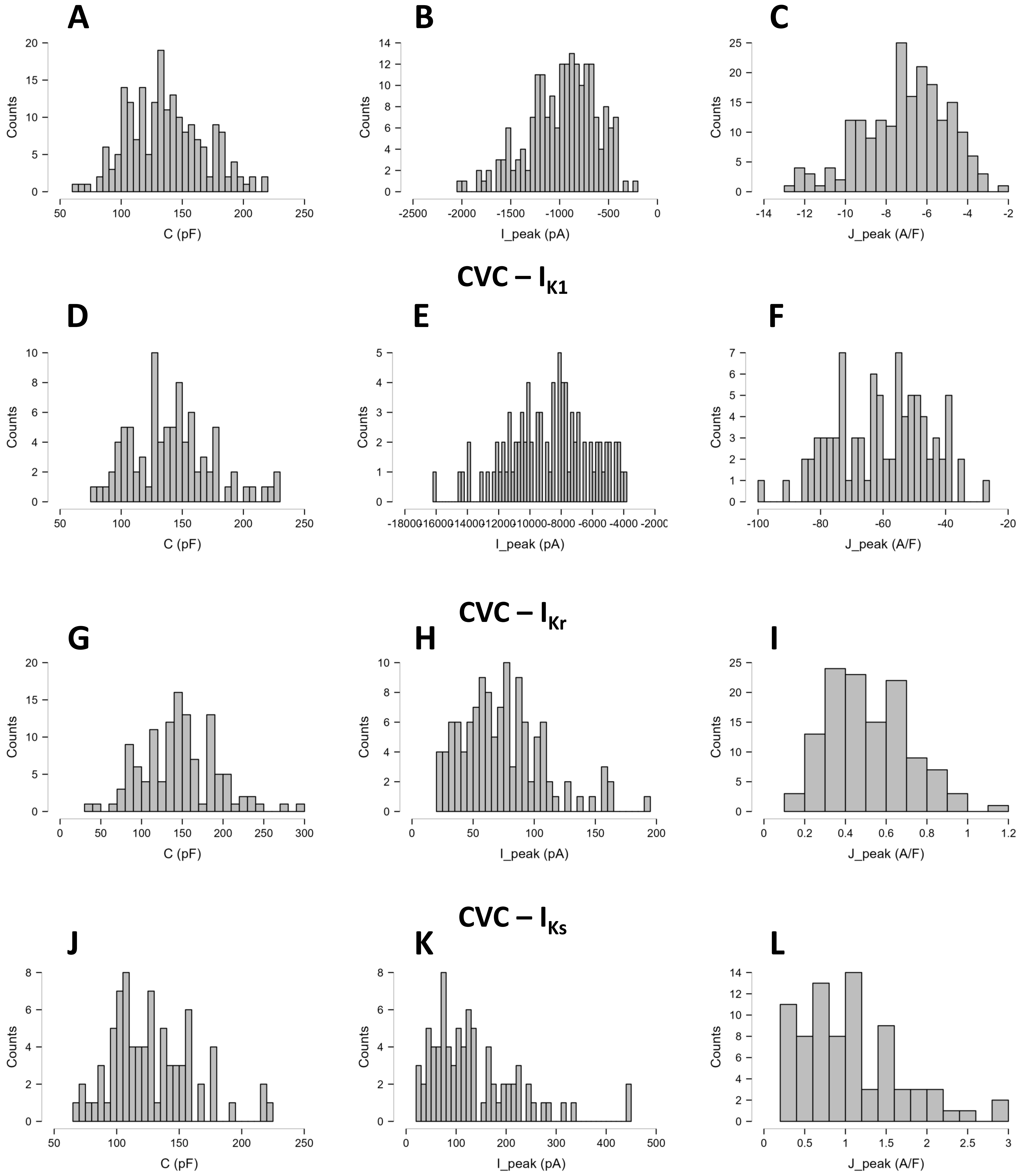

Suppl. Fig. 3.

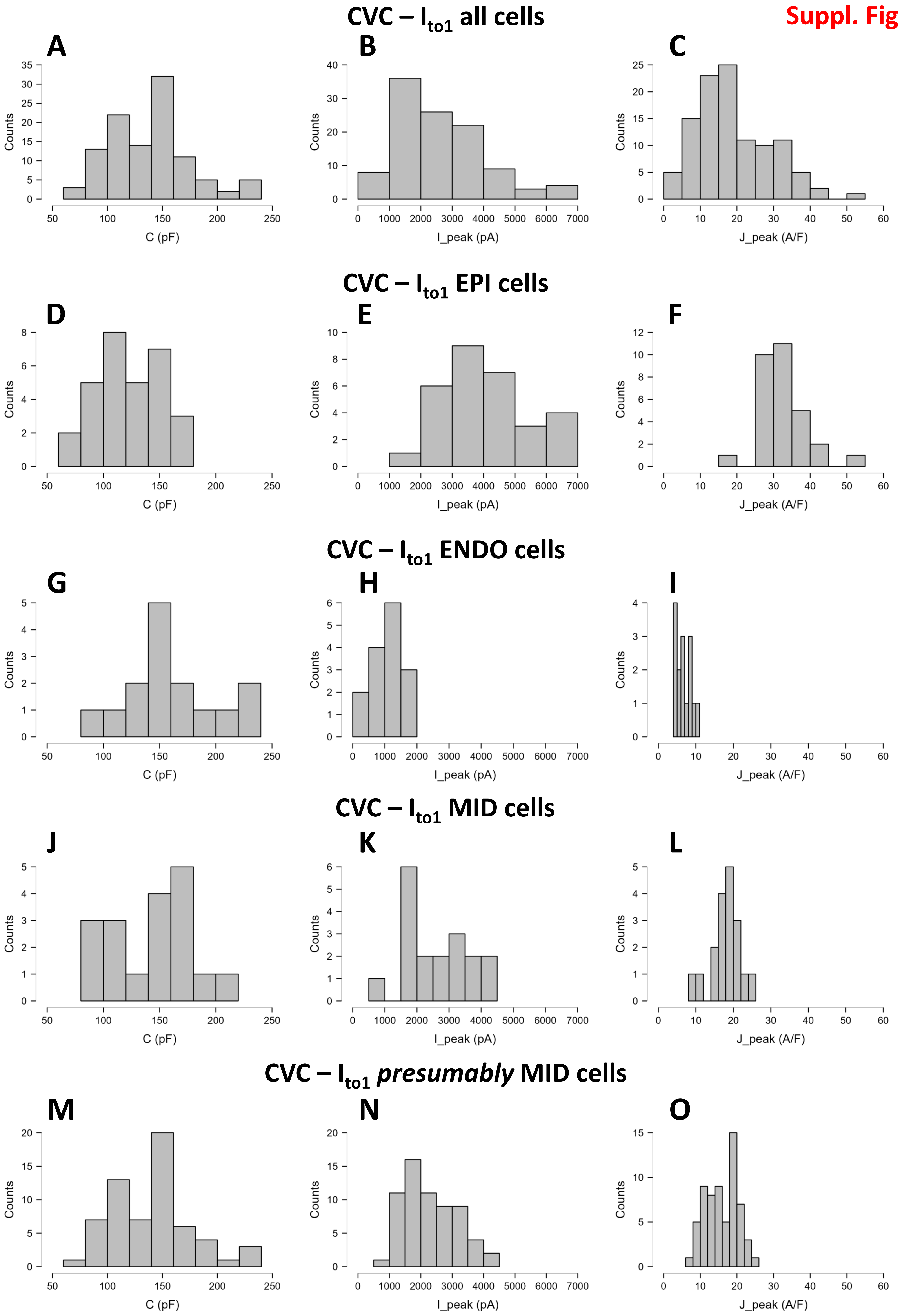

CVC – I_{to1} all cells
A
B
C
CVC – I_{to1} EPI cells
D
E
F
CVC – I_{to1} ENDO cells
G
H
I
CVC – I_{to1} MID cells
J
K
L
CVC – I_{to1} *presumably* MID cells
M
N
O
Counts
C (pF)
I_peak (pA)
J_peak (A/F)

Suppl. Fig. 4.

APVC – $I_{Na,late}$

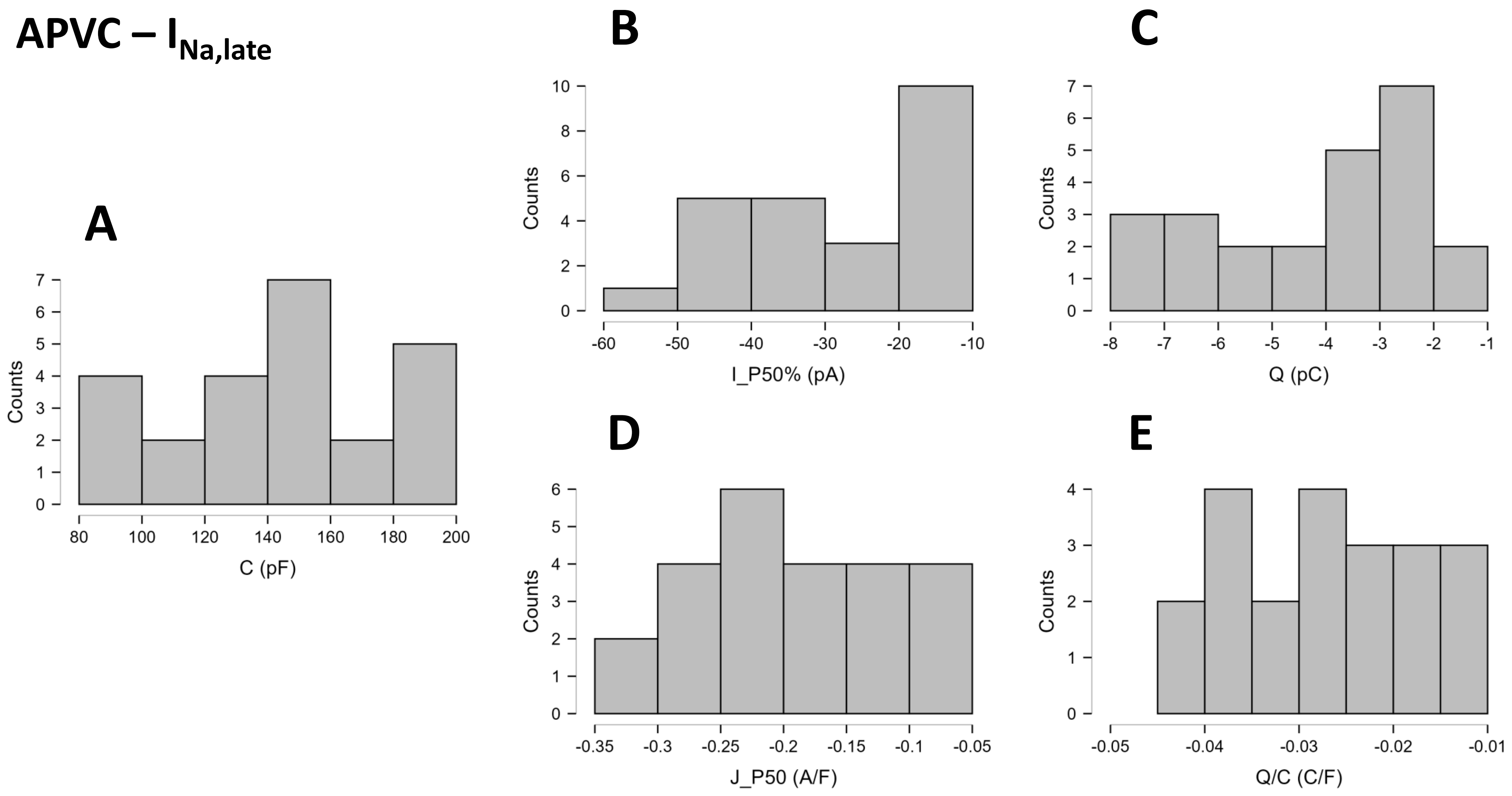

APVC – I_{NCX}

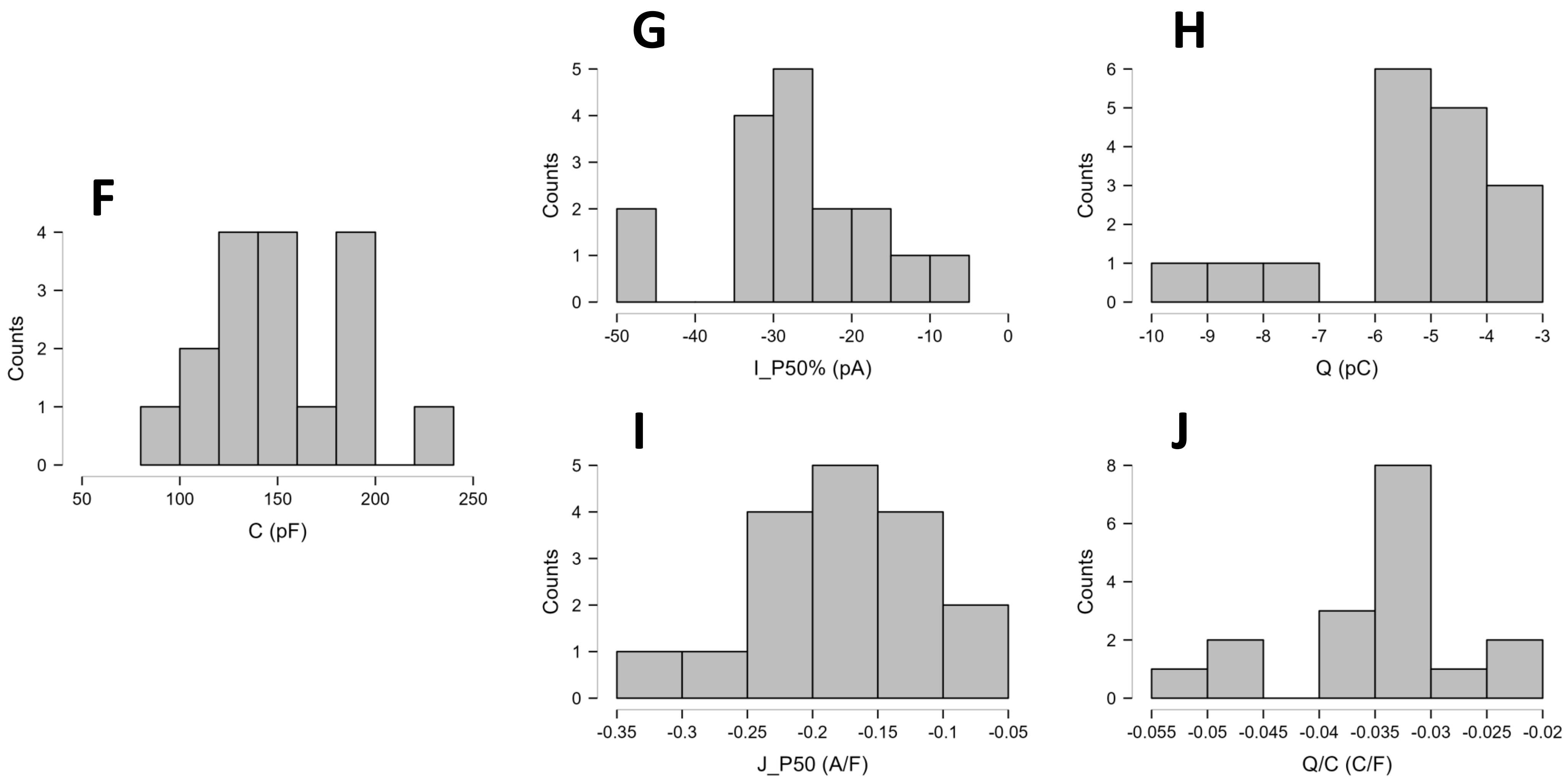

Suppl. Fig. 5.

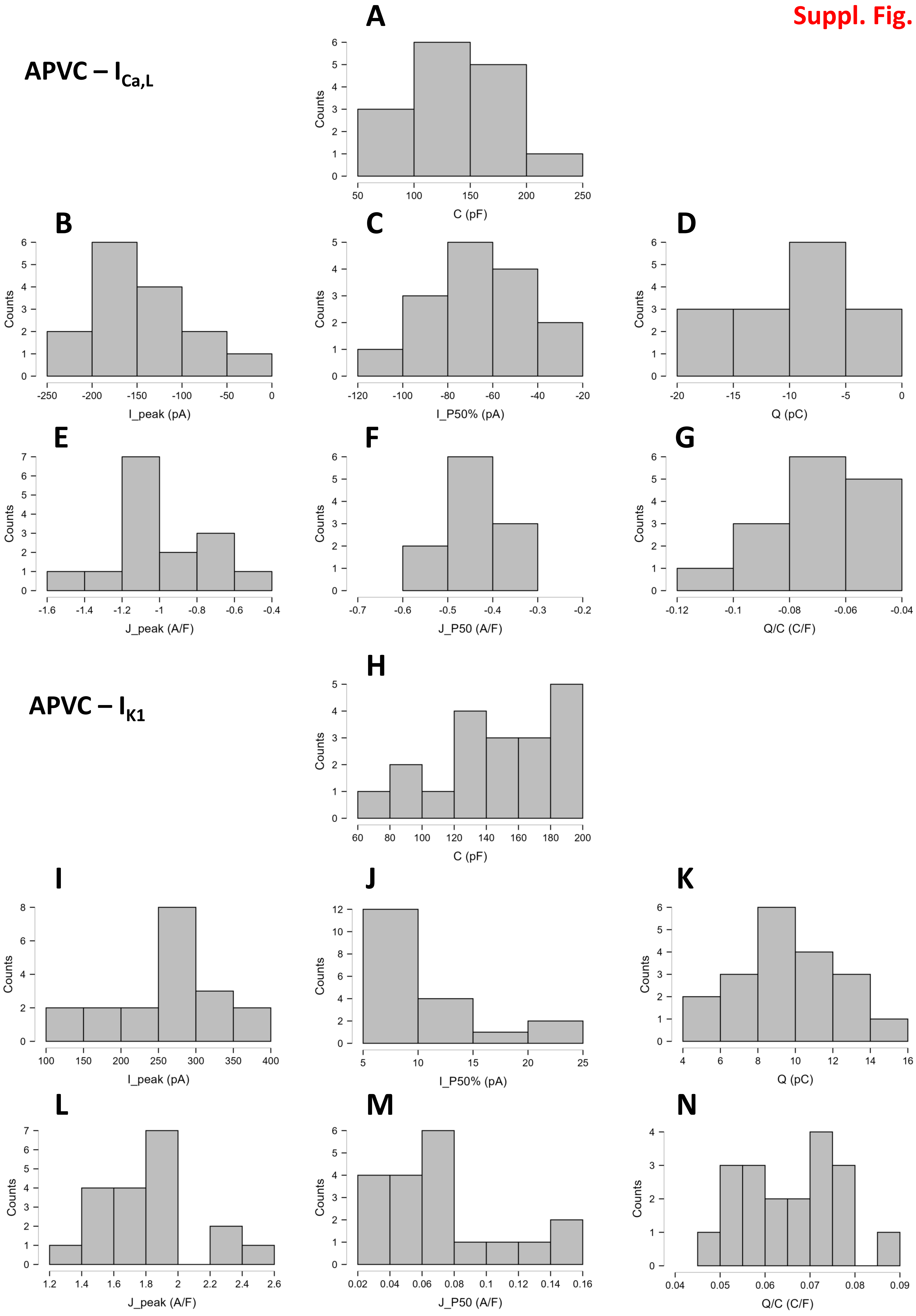
APVC – $I_{Ca,L}$
A
Counts
C (pF)
B
Counts
I_peak (pA)
C
Counts
I_P50% (pA)
D
Counts
Q (pC)
E
Counts
J_peak (A/F)
F
Counts
J_P50 (A/F)
G
Counts
Q/C (C/F)
APVC – I_{K1}
H
Counts
C (pF)
I
Counts
I_peak (pA)
J
Counts
I_P50% (pA)
K
Counts
Q (pC)
L
Counts
J_peak (A/F)
M
Counts
J_P50 (A/F)
N
Counts
Q/C (C/F)

Suppl. Fig. 6.

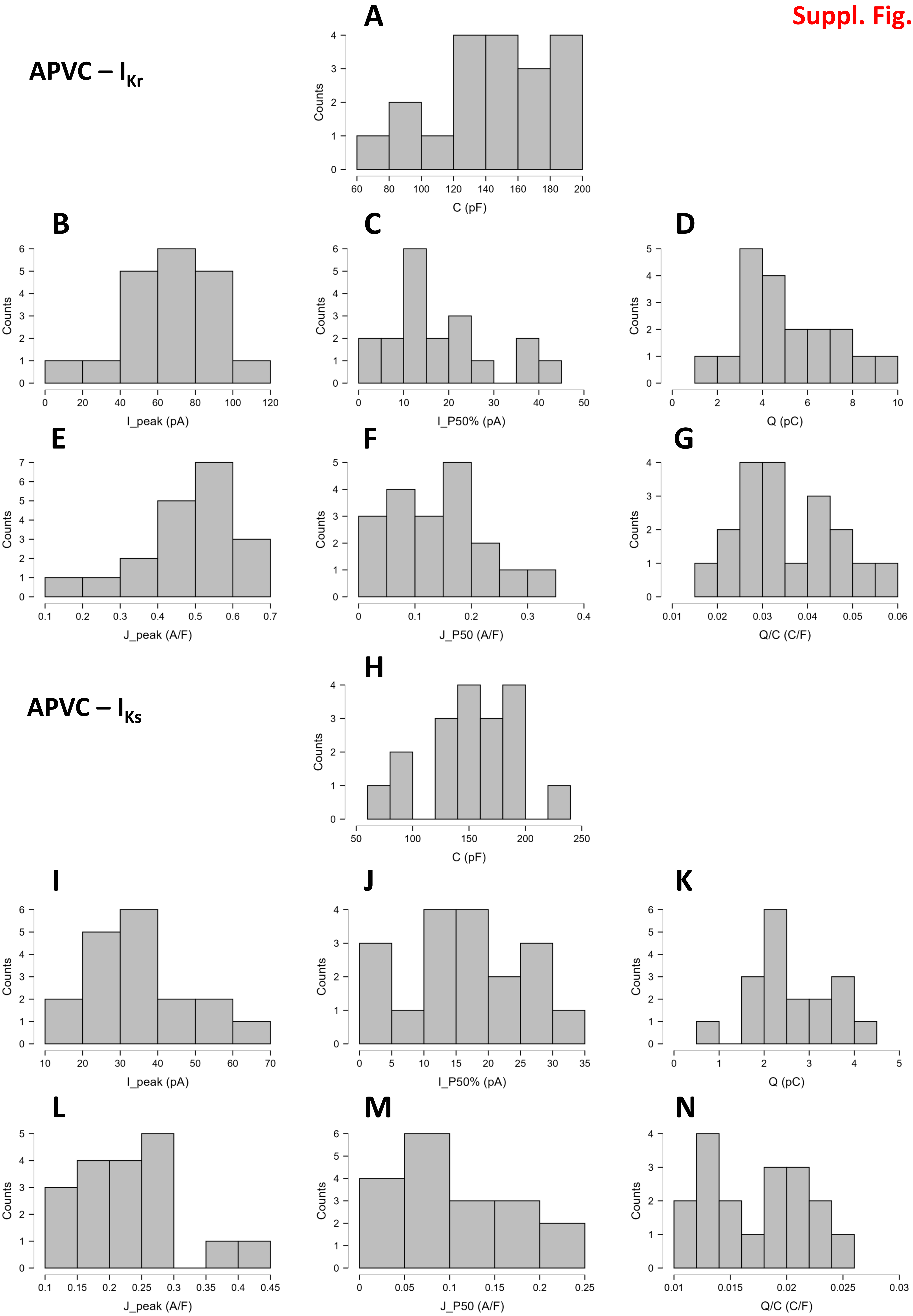
APVC – I_{Kr}
A
Counts
C (pF)
B
Counts
I_peak (pA)
C
Counts
I_P50% (pA)
D
Counts
Q (pC)
E
Counts
J_peak (A/F)
F
Counts
J_P50 (A/F)
G
Counts
Q/C (C/F)
APVC – I_{Ks}
H
Counts
C (pF)
I
Counts
I_peak (pA)
J
Counts
I_P50% (pA)
K
Counts
Q (pC)
L
Counts
J_peak (A/F)
M
Counts
J_P50 (A/F)
N
Counts
Q/C (C/F)
